# Age-related TFEB downregulation in proximal tubules causes systemic metabolic disorders and occasional apolipoprotein A4–related amyloidosis

**DOI:** 10.1172/jci.insight.184451

**Published:** 2024-12-19

**Authors:** Jun Nakamura, Takeshi Yamamoto, Yoshitsugu Takabatake, Tomoko Namba-Hamano, Atsushi Takahashi, Jun Matsuda, Satoshi Minami, Shinsuke Sakai, Hiroaki Yonishi, Shihomi Maeda, Sho Matsui, Hideaki Kawai, Isao Matsui, Tadashi Yamamuro, Ryuya Edahiro, Seiji Takashima, Akira Takasawa, Yukinori Okada, Tamotsu Yoshimori, Andrea Ballabio, Yoshitaka Isaka

**Affiliations:** 1 Department of Nephrology, Osaka University Graduate School of Medicine, Osaka, Japan.; 2 Division of Endocrinology, Diabetes and Metabolism, Beth Israel Deaconess Medical Center and Harvard Medical School, Boston, Massachusetts, USA.; 3 Department of Statistical Genetics and; 4 Department of Respiratory Medicine and Clinical Immunology, Osaka University Graduate School of Medicine, Osaka, Japan.; 5 Laboratory for Systems Genetics, RIKEN Center for Integrative Medical Sciences, Yokohama, Japan.; 6 Department of Medical Biochemistry, Osaka University Graduate School of Medicine, Suita, Osaka, Japan.; 7 Division of Tumor Pathology, Department of Pathology, Asahikawa Medical University, Asahikawa, Japan.; 8 Premium Research Institute for Human Metaverse Medicine (WPI-PRIMe) and; 9 Laboratory of Statistical Immunology, Immunology Frontier Research Center (WPI-IFReC), Osaka University, Suita, Japan.; 10 Department of Genome Informatics, Graduate School of Medicine, the University of Tokyo, Tokyo, Japan.; 11 Health Promotion System Science, Graduate School of Medicine, Osaka University, Suita, Osaka, Japan.; 12 Telethon Institute of Genetics and Medicine (TIGEM), Via Campi Flegrei 34, Pozzuoli, Naples, Italy.; 13 Medical Genetics Unit, Department of Medical and Translational Science, Federico II University, Via Pansini 5, Naples, Italy.; 14 Department of Molecular and Human Genetics, Baylor College of Medicine, Houston, Texas, USA.; 15 Jan and Dan Duncan Neurological Research Institute, Texas Children’s Hospital, Houston, Texas, USA.

**Keywords:** Metabolism, Nephrology, Chronic kidney disease, Fatty acid oxidation, Mitochondria

## Abstract

With the aging of society, the incidence of chronic kidney disease (CKD), a common cause of death, has been increasing. Transcription factor EB (TFEB), the master transcriptional regulator of the autophagy/lysosomal pathway, is regarded as a promising candidate for preventing various age-related diseases. However, whether TFEB in the proximal tubules plays a significant role in elderly patients with CKD remains unknown. First, we found that nuclear TFEB localization in proximal tubular epithelial cells (PTECs) declined with age in both mice and humans. Next, we generated PTEC-specific *Tfeb*-deficient mice and bred them for up to 24 months. We found that TFEB deficiency in the proximal tubules caused metabolic disorders and occasionally led to apolipoprotein A4 (APOA4) amyloidosis. Supporting this result, we identified markedly decreased nuclear TFEB localization in the proximal tubules of elderly patients with APOA4 amyloidosis. The metabolic disturbances were accompanied by mitochondrial dysfunction due to transcriptional changes involved in fatty acid oxidation and oxidative phosphorylation pathways, as well as decreased mitochondrial clearance. This decreased clearance was reflected by the accumulation of mitochondria–lysosome-related organelles, which depended on lysosomal function. These results shed light on the presumptive mechanisms of APOA4 amyloidosis pathogenesis and provide a therapeutic strategy for CKD-related metabolic disorders and APOA4 amyloidosis.

## Abstract 2

With age, decreased TFEB activity in proximal tubules causes systemic metabolic disorders and sometimes apolipoprotein A4-related amyloidosis through combined mitochondrial and lysosomal dysfunction.

## Graphical abstract

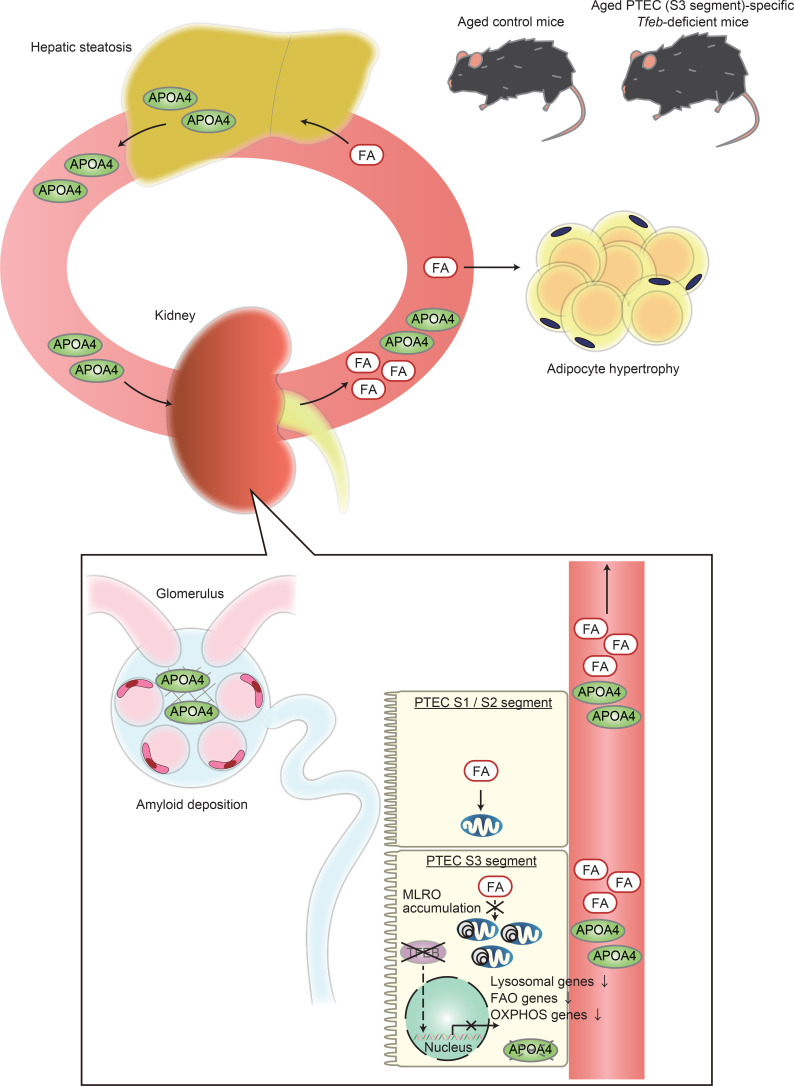

## Introduction

Chronic kidney disease (CKD), defined as a persistent decrease in kidney function or abnormalities of kidney structure for at least 3 months, affects approximately 10% of the population worldwide (1). CKD is one of the leading causes of death from noncommunicable diseases, and the number of CKD-associated deaths has been increasing among the elderly in recent years (2, 3). Sodium–glucose cotransporter 2 inhibitors have demonstrated a markedly beneficial effect on CKD in individuals with or without diabetes, and this effect has been shown to extend to the elderly population (4). However, there remains a marginal risk of CKD progression, indicating the need for further treatment options.

With the aging of society, the incidence of various age-related diseases, such as CKD, has been increasing. To combat these diseases, a variety of treatment methods have been identified, including autophagy modulators, stem cell therapy, antioxidative treatments, and elimination of senescent cells with senolytics (5, 6). Notably, a clinical trial of senolytics is currently being conducted in patients with diabetic kidney disease (7). However, it is important to recognize that senescence plays a crucial role in slowing tumor progression (8), and continuous or acute elimination of senescent cells can lead to health deterioration (9). Therefore, strategies to suppress cellular senescence seem to be safe and promising.

Interventions that properly regulate autophagy, an intracellular degradation system essential for cellular homeostasis and adaptation to stress, can prevent age-related diseases (10). We have demonstrated that autophagy in proximal tubular epithelial cells (PTECs) protects against acute kidney injury (11–13) and prevents CKD progression under various chronic conditions, including kidney aging (14–20).

Transcription factor EB (TFEB), the master transcriptional regulator of the autophagy/lysosomal pathway (21, 22), is regarded as a promising candidate for the prevention of various age-related diseases and extension of lifespan (23–25). This is supported by the fact that TFEB regulates mitochondrial function (26–29), in addition to the autophagy/lysosomal pathway, and that dysfunction of both pathways is a hallmark of aging (30). Furthermore, it has been reported that MondoA and HKDC1, both of which are closely related to TFEB, are candidates for preventing cellular senescence (31–33). TFEB is also considered to be a therapeutic target in kidney diseases (34), and we have clarified that TFEB in PTECs protects against kidney injury by maintaining lysosomal homeostasis (35, 36).

Given this background, we hypothesize that TFEB plays an important role in kidney aging, especially in PTECs, but this remains to be elucidated. In this study, PTEC-specific *Tfeb*-deficient mice were bred for up to 24 months, and the role of TFEB was examined using various methods including single-cell RNA-Seq (scRNA-Seq) analysis and electron microscopy.

## Results

### Nuclear TFEB localization in PTECs is downregulated with age.

**Figure 1:**
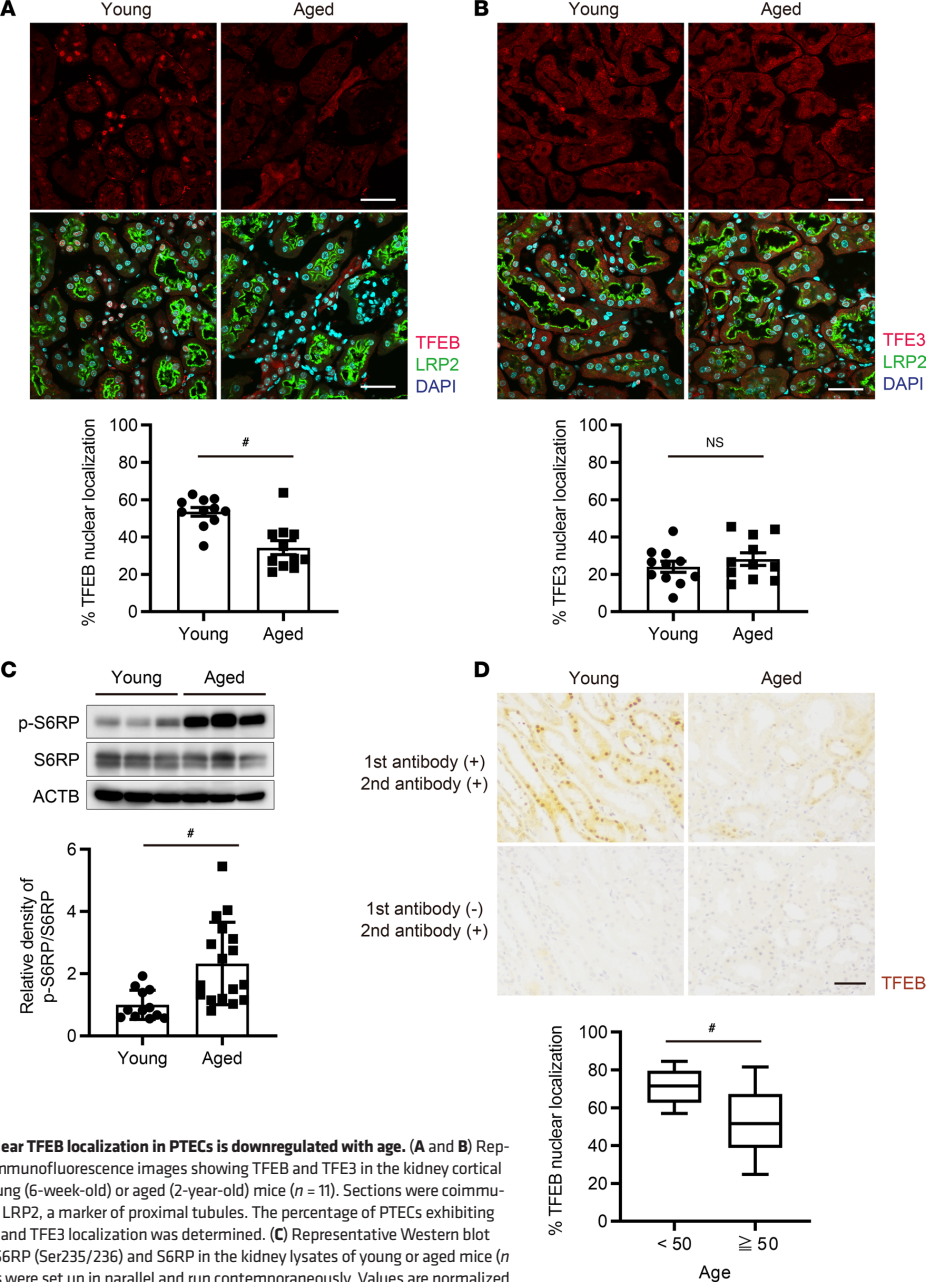
Nuclear TFEB localization in PTECs is downregulated with age. (**A** and **B**) Representative immunofluorescence images showing TFEB and TFE3 in the kidney cortical regions of young (6-week-old) or aged (2-year-old) mice (*n* = 11). Sections were coimmunostained for LRP2, a marker of proximal tubules. The percentage of PTECs exhibiting nuclear TFEB and TFE3 localization was determined. (**C**) Representative Western blot images of p-S6RP (Ser235/236) and S6RP in the kidney lysates of young or aged mice (*n* = 12–17). Blots were set up in parallel and run contemporaneously. Values are normalized by the mean value of young mice. (**D**) Representative images of IHC staining for TFEB on kidney specimens obtained from young (younger than 50 years old) or aged (older than 50 years old) patients. Specimens were counterstained with hematoxylin. Kidney biopsy samples from patients with CKD were analyzed, using the dataset previously reported in our papers, with modifications (35) (*n* = 24). Scale bars: 40 μm (**A** and **B**) and 50 μm (**D**). Data are shown bar graphs, showing mean ± SEM or box plots showing median values and interquartile ranges. Boxes represent the 25th and 75th percentiles, lines inside the boxes represent medians, and whiskers are plotted by Tukey method. ^#^*P* < 0.05 versus young littermates or young patients (**A**–**C**, 2-tailed Student’s *t* test; **D**, Wilcoxon rank-sum test).

WT mice aged 6 weeks or 24 months were compared, representing young and aged mice, respectively. An immunofluorescence study demonstrated that nuclear TFEB localization in PTECs was significantly decreased in aged mice compared with young mice (Figure 1A and Supplemental Figure 1A; supplemental material available online with this article; https://doi.org/10.1172/jci.insight.184451DS1). On the other hand, Transcription factor binding to IGHM enhancer 3 (TFE3), another member of the microphthalmia/transcription factor E (MiTF/TFE) family, did not change with age (Figure 1B). Aging and the mTOR signaling are closely linked (37), and TFEB is phosphorylated and retained in the cytoplasm by mTOR complex 1 (mTORC1) (38). The phosphorylation of S6 Ribosomal Protein (S6RP), a downstream target of mTORC1, was increased in the aged kidneys (Figure 1C), indicating that activated mTORC1 signaling is one of the reasons for decreased nuclear TFEB localization. Additionally, we analyzed data on kidney biopsy samples from patients with CKD, using the dataset reported in our previous studies, with modifications (20, 35). Compared with nonobese young patients, older patients exhibited markedly decreased nuclear TFEB localization, mirroring the findings observed in aged mice (Figure 1D, Supplemental Figure 1B, and Supplemental Tables 1 and 2). Collectively, these results indicate that nuclear TFEB localization in PTECs decreases with age.

### Long-term TFEB deficiency in PTECs does not largely induce renal dysfunction.

We generated transgenic mice that specifically overexpressed TFEB in the proximal tubules to elucidate the role of TFEB during the aging process. The *Tfeb* conditional overexpressing mouse line, carrying *Tfeb-3xFlag^fs/fs^* under the control of a strong chicken β-actin (CAG) promoter (22), was crossed with the kidney androgen-regulated protein–Cre (KAP-Cre) mouse line expressing Cre recombinase almost exclusively in the kidney and specifically in the S3 segment of the proximal tubules (11, 39, 40). A significant increase in kidney weight was observed in PTEC-specific *Tfeb*-overexpressing mice at 4 months of age (Supplemental Figure 1C). Histological analysis showed aberrant cell proliferation and vacuolar degeneration in proximal tubules (Supplemental Figure 1D). Notably, in a previous report, other kidney-specific *Tfeb*-overexpressing mice generated by crossing the Cadherin16-Cre mouse line displayed massive proliferation of cells in collecting ducts and distal tubules, resulting in tumorigenesis (41). This raised concerns about maintaining PTEC-specific *Tfeb*-overexpressing mice up to 24 months of age.

To explore the role of TFEB in aging PTECs, we compared PTEC-specific *Tfeb*-deficient *Tfeb^fl/fl^* KAP mice with *Tfeb^fl/fl^* mice for up to 24 months. An immunofluorescence study demonstrated that TFEB was specifically deleted in the S3 segment of the proximal tubules (Supplemental Figure 2). In the majority of *Tfeb^fl/fl^* KAP mice, periodic acid–Schiff (PAS) staining indicated minimal morphological abnormalities, including tumorigenesis, in the proximal tubules of both the aged groups (Supplemental Figure 3A). Additionally, Picrosirius red staining showed little enhancement of interstitial fibrosis (Supplemental Figure 3B). Consistent with these staining results, kidney function assessed by blood tests exhibited no significant differences between the 2 aged groups (Supplemental Figure 3C). Together, these results suggest that long-term TFEB deficiency in PTECs does not significantly affect morphological features in the kidney.

### TFEB downregulation in PTECs causes apolipoprotein A4 (APOA4) amyloidosis in aged mice.

**Figure 2:**
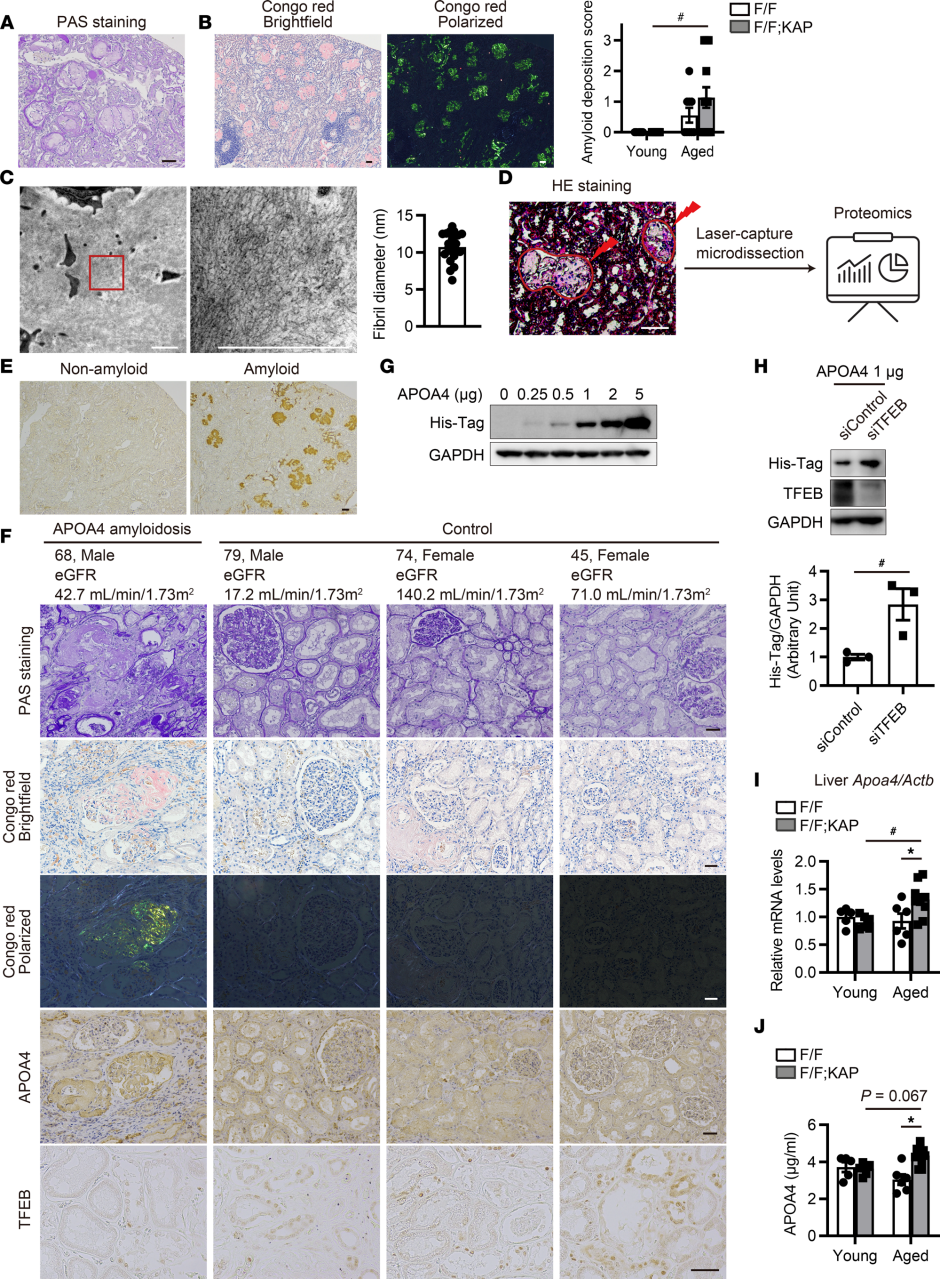
TFEB downregulation in PTECs causes apolipoprotein A4 (APOA4) amyloidosis with age. (**A**, **B**, and **E**) Representative images of PAS staining (**A**), Congo red staining (**B**), and APOA4 immunostaining (**E**) in the kidneys of aged *Tfeb^fl/fl^* KAP mice with (**A**, **B**, and **E**) or without (**E**) amyloid deposits. (**B**) Amyloid deposition scores (*n* = 9–15). (**C**) Representative electron micrographs of the amyloid deposits. Fibril diameters were measured (*n* = 20). (**D**) Schematic illustration of the LMD–LC-MS/MS procedure. The glomeruli with amyloid deposits were isolated and processed for proteomics. (**F**) Representative images from the cortical regions of human autopsy kidney samples. (**G** and **H**) Representative Western blot images from HK-2 cells transfected with (**H**) or without (**G**) TFEB siRNA after treatment with recombinant human APOA4 protein (with His-Tag) for 3 hours (*n* = 3). This experiment was repeated 3 times. Blots were set up in parallel and run contemporaneously (**H**). (**I**) *Apoa4* mRNA levels relative to *Actb* in the livers of mice (*n* = 5–10). (**J**) APOA4 concentrations in the plasma of mice (*n* = 5–10). Scale bars: 50 μm (**A**, **B**, and **D**–**F**) and 1 μm (**C**). Data are shown bar graphs, showing mean ± SEM. Values are normalized by the mean value of HK-2 cells transfected with siControl (**H**) or young *Tfeb^fl/fl^* mice (**I**). *P < 0.05 versus age-matched *Tfeb^fl/fl^* control littermates or siControl HK-2 cells; ^#^*P* < 0.05 versus young mice or siControl HK-2 cells (**B**, **I**, and **J**, 1-way ANOVA followed by the Tukey-Kramer test; **H**, 2-tailed Student’s *t* test). Sections were counterstained with hematoxylin. Bright-field images of Congo red staining were captured along with corresponding apple-green birefringence under polarized light. F/F, *Tfeb^fl/fl^* mice; F/F;KAP, *Tfeb^fl/fl^* KAP mice; eGFR, estimated glomerular filtration rate.

We identified that distinctive extracellular, amorphous, and hypocellular material was prominently deposited in the glomeruli and in the interstitium in several aged *Tfeb^fl/fl^* KAP mice (Figure 2A). Of these, 26.7% (4 of 15) showed extensive deposition as indicated by a score of “3 (extensive)” for amyloid deposition. This suggested the presence of amyloid fibril deposits (Figure 2B). Congo red staining, the gold standard for identifying amyloid fibrils (42), revealed these deposits with characteristic red-orange staining and apple-green birefringence under polarized light (Figure 2B). In line with these results, albuminuria was detected in aged *Tfeb^fl/fl^* KAP mice (Supplemental Figure 3D). Additionally, electron microscopy demonstrated fibrils with a mean diameter of 10 nm, consistent with amyloid fibrils (Figure 2C) (42).

Next, we isolated glomeruli with amyloid deposits by performing laser-capture microdissection (LMD) from formalin-fixed paraffin-embedded (FFPE) samples and identified amyloidosis-related protein using liquid chromatography–tandem mass spectrometry–based (LC-MS/MS–based) proteomics (Figure 2D), hereinafter referred to as LMD–LC-MS/MS (43, 44). This LMD–LC-MS/MS analysis revealed the presence of amyloid signature proteins, including apolipoprotein E, clusterin, and vitronectin, indicating amyloid deposition and suggesting potential identification of causative amyloidogenic proteins from this sample. A representative list of proteins known to cause or be associated with amyloidosis is shown in Table 1. Among these known amyloidogenic proteins, APOA4 exhibited the highest spectral counts in this study, followed by APOA2 and serum amyloid A (SAA) protein. While age-related APOA2 amyloidosis has been reported in aged mice — and is characterized by amyloid deposits, especially in the intestines (45) — only minor amyloid deposits were observed in the intestines (Supplemental Figure 4A). Immunoglobulin heavy and light chains were also detected, but no amyloid deposits were identified in the bone marrow (Supplemental Figure 4A) even though most patients with immunoglobulin-related amyloidosis exhibit bone marrow amyloid deposits (46). In addition to the kidney, amyloid deposits were also detected in the interstitium of the heart and the vessel walls of the liver (Supplemental Figure 4A). The result of LMD–LC-MS/MS analysis and these characteristics resemble those of mice with APOA4 amyloidosis in the absence of DREAM (Dp, Rb-like, E2F, and MuvB) assembly (47), indicating that APOA4 was the most likely cause of amyloidosis. Indeed, staining for APOA4 was highly positive, corresponding to the distribution of Congo red^+^ regions (Figure 2E and Supplemental Figure 4B). Furthermore, we investigated the relationship between APOA4 amyloidosis and nuclear TFEB localization in PTECs using human autopsy samples. Compared with control young patients, nuclear TFEB localization in PTECs was reduced in the elderly patient withAPOA4 amyloidosis with a genetic variant of APOA4, which we previously reported (48) (Figure 2F and Supplemental Figure 4C). This result may indicate that decreased nuclear TFEB localization with age triggers APOA4 amyloidosis in a patient with a genetic variant of APOA4.

We next investigated the reasons for the increase in APOA4. First, previous studies have suggested that APOA4 may be degraded in the lysosomes of PTECs (49, 50), although the exact mechanism is not well understood. To investigate this further, we used HK-2 cells (human PTECs) treated with recombinant human APOA4 and assessed whether TFEB, a master regulator of lysosomal function, regulates APOA4 accumulation. We confirmed that HK-2 cells showed an increase expression of APOA4 in a dose-dependent manner (Figure 2G). As expected, TFEB knockdown increased APOA4 accumulation (Figure 2H), indicating that APOA4 regulation depends on TFEB in PTECs. In agreement with this result, PTECs in aged *Tfeb^fl/fl^* KAP mice showed increased APOA4 deposition (Supplemental Figure 4D). Second, we assessed the extent of APOA4 in the intestine and the liver because APOA4 is mainly synthesized by these organs (51). APOA4 was significantly increased in the livers of aged *Tfeb^fl/fl^* KAP mice (Figure 2I and Supplemental Figure 4, E and F). Consistent with these findings, plasma APOA4 was also increased in aged *Tfeb^fl/fl^* KAP mice (Figure 2J). These data suggest that APOA4 amyloidosis in aged *Tfeb^fl/fl^* KAP mice may be induced by both decreased APOA4 degradation in PTECs and increased hepatic APOA4 synthesis.

### TFEB deficiency in PTECs leads to metabolic disorders in aged mice.

**Figure 3:**
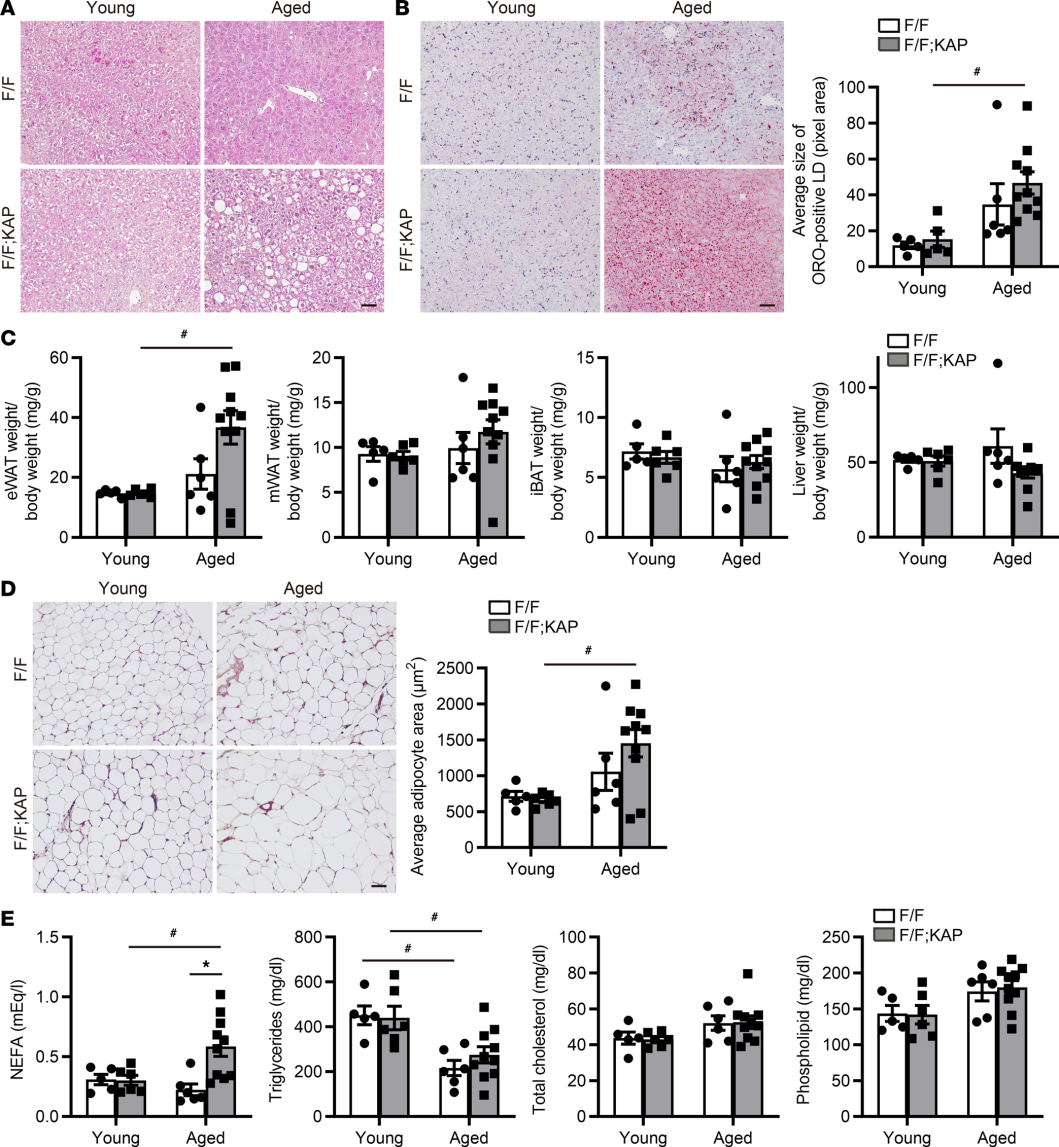
TFEB deficiency in PTECs leads to metabolic disorders in aged mice. (**A** and **B**) Representative images of H&E staining (**A**) and Oil red O (ORO) staining (**B**) in the livers of young and aged *Tfeb^fl/fl^* or *Tfeb^fl/fl^* KAP mice (*n* = 5–10). The size of ORO^+^ lipid droplets (LDs) is measured. (**C**) The ratio of the organ weight of epididymal white adipose tissue (eWAT), mesenteric WAT (mWAT), liver, and interscapular brown adipose tissue (iBAT) to the total body weight of mice (*n* = 5–10). (**D**) Representative images of H&E staining and adipose cell size in eWAT of mice (*n* = 5–10). (**E**) Plasma nonesterified fatty acid (NEFA), triglycerides, total cholesterol, and phospholipid levels of mice (*n* = 5–10). Scale bars: 50 μm (**A**, **B**, and **D**). Data are shown bar graphs, showing mean ± SEM. *P < 0.05 versus age-matched *Tfeb^fl/fl^* control littermates; ^#^*P* < 0.05 versus young mice (**B**–**E**, 1-way ANOVA followed by the Tukey-Kramer test). F/F, *Tfeb^fl/fl^* mice; F/F;KAP, *Tfeb^fl/fl^* KAP mice.

In mice, hepatic steatosis induces APOA4 expression, which reduces the lipid burden (52). Histological evaluation revealed that the degree of hepatic steatosis was more severe in aged *Tfeb^fl/fl^* KAP mice than in aged *Tfeb^fl/fl^* mice (Figure 3, A and B). Therefore, we investigated whether systemic metabolic changes were evident in aged *Tfeb^fl/fl^* KAP mice. At around 16 months of age, *Tfeb^fl/fl^* KAP mice exhibited a lower rate of body weight loss compared with *Tfeb^fl/fl^* mice, although the difference was not significant (Supplemental Figure 5, A and B). Consistent with this observation, epididymal white adipose tissue (eWAT) demonstrated greater weight and adipose cell size in aged *Tfeb^fl/fl^* KAP mice (Figure 3, C and D). Moreover, TFEB deficiency in PTECs increased the expression of adipogenic genes in eWAT, including *Acaca* and *Fasn*, and the expression of *Plin2*, a gene that participates in lipid droplet (LD) formation (53) (Supplemental Figure 5C). In addition, aged *Tfeb^fl/fl^* KAP mice showed increased plasma levels of circulating free fatty acids (FFAs) (Figure 3E). Taken together, our findings indicate that the loss of TFEB in PTECs causes metabolic disorders in aged mice.

### scRNA-Seq analyses identify downregulation of the oxidative phosphorylation pathway with age, caused by TFEB deficiency in the S3 segment.

**Figure 4:**
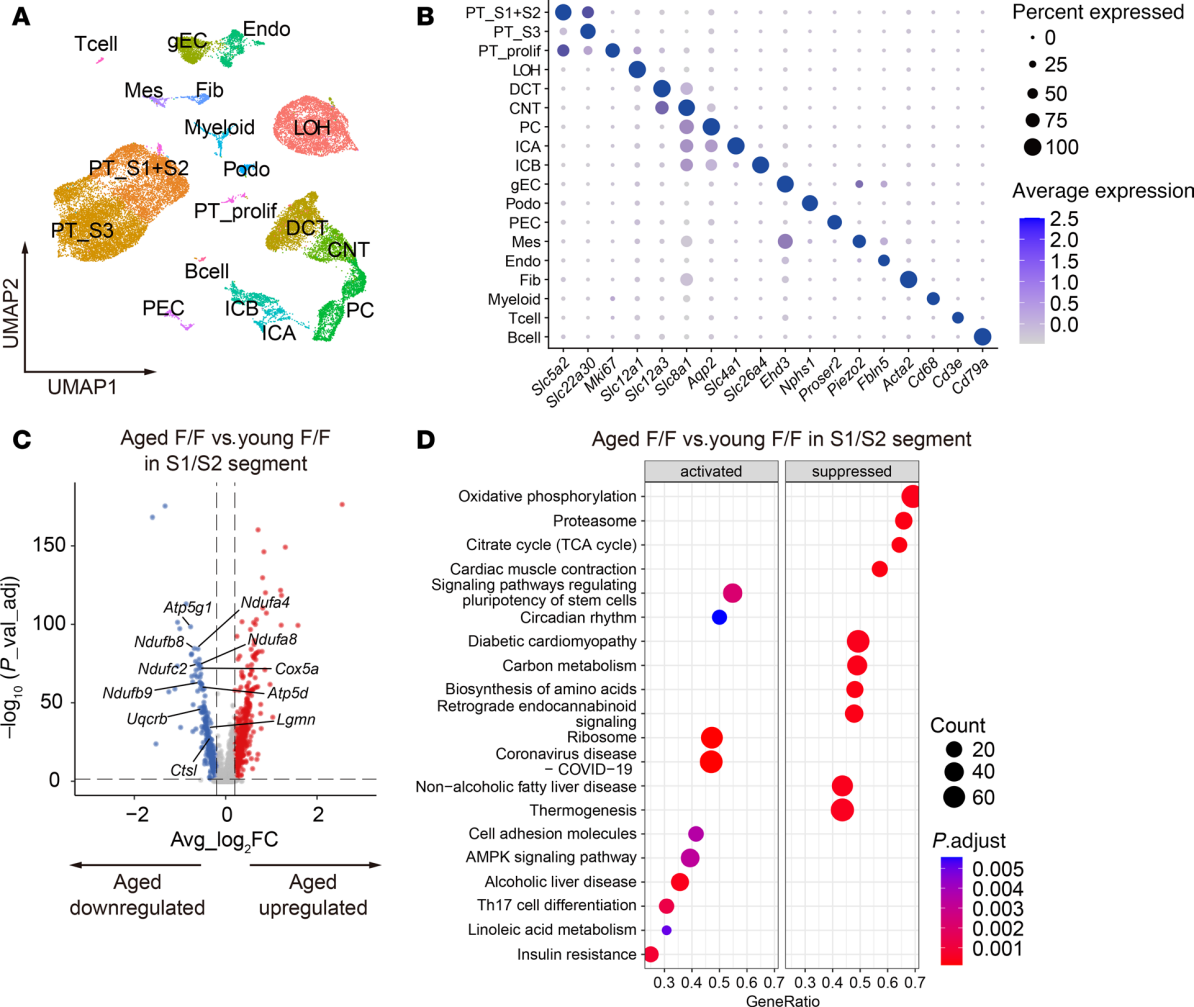
scRNA-Seq analyses capture the characteristics of kidney aging. (**A**) Kidneys from the young and aged *Tfeb^fl/fl^* or *Tfeb^fl/fl^* KAP mice (*n* = 2–3) were subjected to scRNA-Seq analyses. Cells were mapped by UMAP. Cells derived from 4 groups are shown as integrated figure. (**B**) Dot plot showing the expression of cell type marker genes across the clusters. Dot size denotes the percentage of cells expressing the marker genes. Color scale represents average gene expression values. (**C**) Volcano plots depicting differentially expressed genes. Colored dots correspond to individual genes whose expression levels were downregulated (blue) and upregulated (red). (**D**) Results of KEGG pathway GSEA. The dot size represents the numbers of genes. The dot color scale corresponds to the adjusted *P* value. PT_S1+S2, S1/S2 segments of proximal tubule; PT_S3, S3 segment of proximal tubule; PT_prolif, proliferative proximal tubule; LOH, loop of Henle; DCT, distal convoluted tubule; CNT, connecting tubule; PC, principal cell; ICA, type A intercalated cell; ICB, type B intercalated cell; gEC, glomerular endothelial cell; Podo, podocyte; PEC, glomerular parietal epithelial cell; Mes, mesangial cell; Endo, extraglomerular endothelial cell; Fib, fibroblast; p_val_ad, adjusted *P* value; Avg_log_2_FC, average log_2_ fold change; F/F, *Tfeb^fl/fl^* mice.

To elucidate the mechanisms underlying the metabolic disorders in aged *Tfeb^fl/fl^* KAP mice, we performed scRNA-Seq analyses of the kidneys from young or aged *Tfeb^fl/fl^* mice and *Tfeb^fl/fl^* KAP mice. We classified the constituent cells into 18 clusters on the basis of cell type marker genes, with a specific focus on the proximal tubule cluster (Figure 4, A and B, and Supplemental Figure 6A). First, we analyzed the S1/S2 segment clusters of young and aged *Tfeb^fl/fl^* mice to confirm the effect of aging. In the aged *Tfeb^fl/fl^* mice, 586 genes were significantly upregulated, and 377 genes were downregulated (Figure 4C and Supplemental Table 3). Kyoto Encyclopedia of Genes and Genomes (KEGG) pathway enrichment analyses revealed the suppression of the oxidative phosphorylation (OXPHOS), citrate cycle (TCA cycle), and thermogenesis pathways in the S1/S2 segment clusters of aged mice (Figure 4D), whereas these pathways were not suppressed in the S3 segment clusters (Supplemental Figure 6, B and C, and Supplemental Table 4). This corroborates that the scRNA-Seq analyses adequately captured the characteristics of kidney aging, as mitochondrial dysfunction is a hallmark of aging (30).

**Figure 5:**
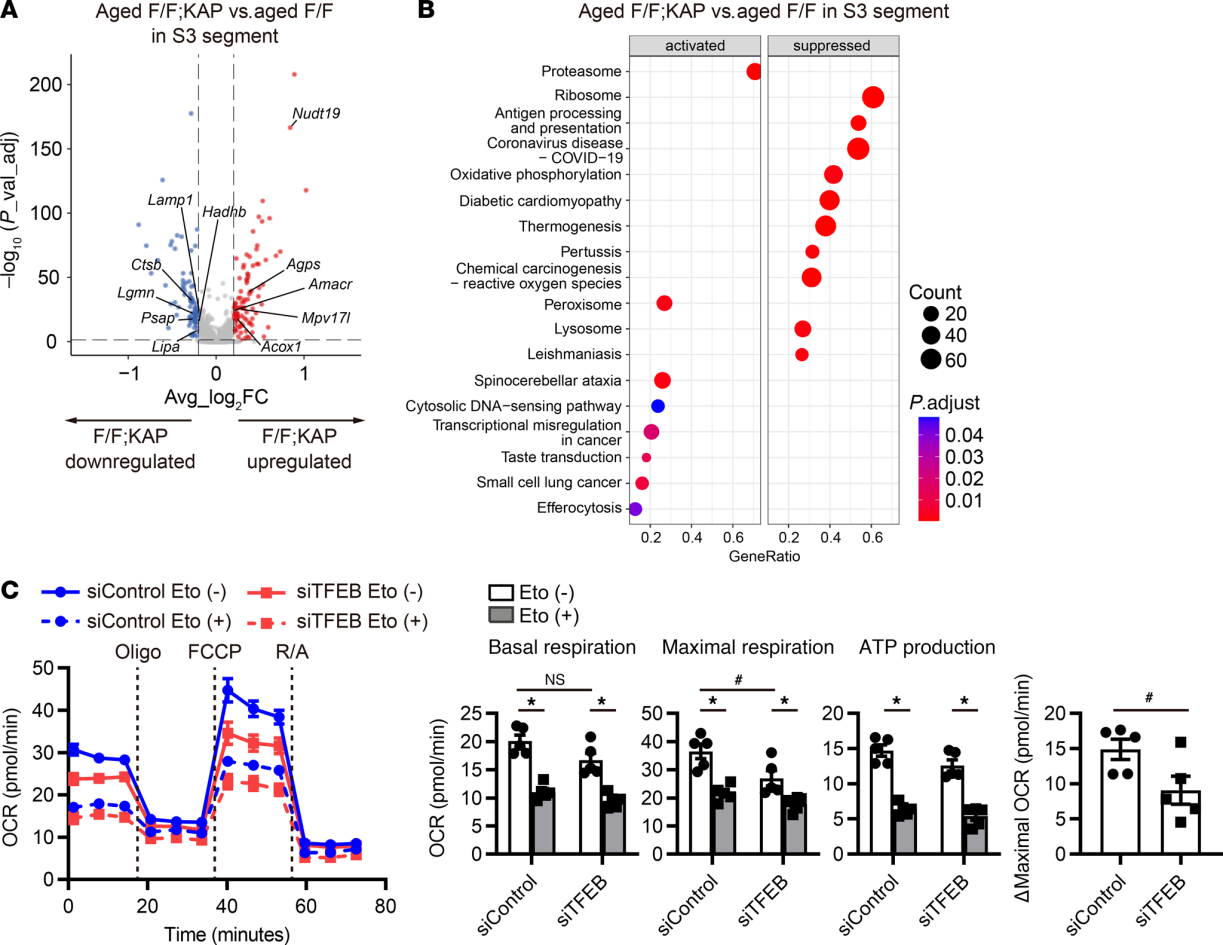
TFEB deficiency causes downregulation of the oxidative phosphorylation pathway in the S3 segment with age. (**A**) Volcano plots depicting differentially expressed genes. Colored dots correspond to individual genes whose expression levels were downregulated (blue) and upregulated (red). (**B**) Results of KEGG pathway GSEA. The dot size represents the numbers of genes. The dot color scale corresponds to the adjusted *P* value. (**C**) The OCR profiles of the HK-2 cells transfected with TFEB siRNA. Measurements were performed after addition of 40 μM etomoxir for 1 hour (*n* = 5). This experiment was repeated 3 times. Data are shown bar graphs, showing mean ± SEM. *P < 0.05 versus Eto-untreated HK-2 cells; ^#^*P* < 0.05 versus siControl HK-2 cells (1-way ANOVA followed by the Tukey-Kramer test or 2-tailed Student’s *t* test). p_val_ad, adjusted *P* value; Avg_log_2_FC, average log_2_ fold change; F/F, *Tfeb^fl/fl^* mice; F/F;KAP, *Tfeb^fl/fl^* KAP mice; Eto, etomoxir; Oligo, oligomycin; R/A, rotenone/antimycin A.

Next, to investigate the effect of TFEB deficiency in the S3 segment of aged mice, we compared the S3 segment cluster between aged *Tfeb^fl/fl^* KAP mice and aged *Tfeb^fl/fl^* mice. The lysosomal pathway is the representative pathway controlled by TFEB (22). A volcano plot showed that several lysosomal genes, including *Ctsb* and *Lamp1,* were downregulated in the S3 segment cluster of aged *Tfeb^fl/fl^* KAP mice (Figure 5A and Supplemental Table 5). KEGG pathway enrichment analyses also demonstrated that the lysosomal pathway was downregulated in the S3 segment cluster of aged *Tfeb^fl/fl^* KAP mice, although TFEB deficiency in the S3 segment did not affect this pathway in young mice (Figure 5B and Supplemental Figure 6, D and E). Moreover, the OXPHOS and thermogenesis pathway were suppressed in aged *Tfeb^fl/fl^* KAP mice, but not in young *Tfeb^fl/fl^* KAP mice (Supplemental Figure 6F). TFEB also controls the expression of genes involved in mitochondrial biogenesis, fatty acid oxidation (FAO), and OXPHOS through peroxisome proliferator–activated receptor γ coactivator 1 α–mediated (PPARGC1α-mediated) and PPARα-mediated pathways, as well as pathways not mediated by these proteins (29, 54). These changes indicate an increased dependence on TFEB during kidney aging. Finally, we monitored cellular oxygen consumption rates (OCRs) with an extracellular flux analyzer, using control and TFEB knockdown HK-2 cells. Maximal OCR was decreased in the TFEB knockdown cells (Figure 5C). These results underscore that TFEB deficiency downregulates OXPHOS in the proximal tubules of aged mice.

### TFEB deficiency reduces mitochondrial clearance in the S3 segment with age.

**Figure 6:**
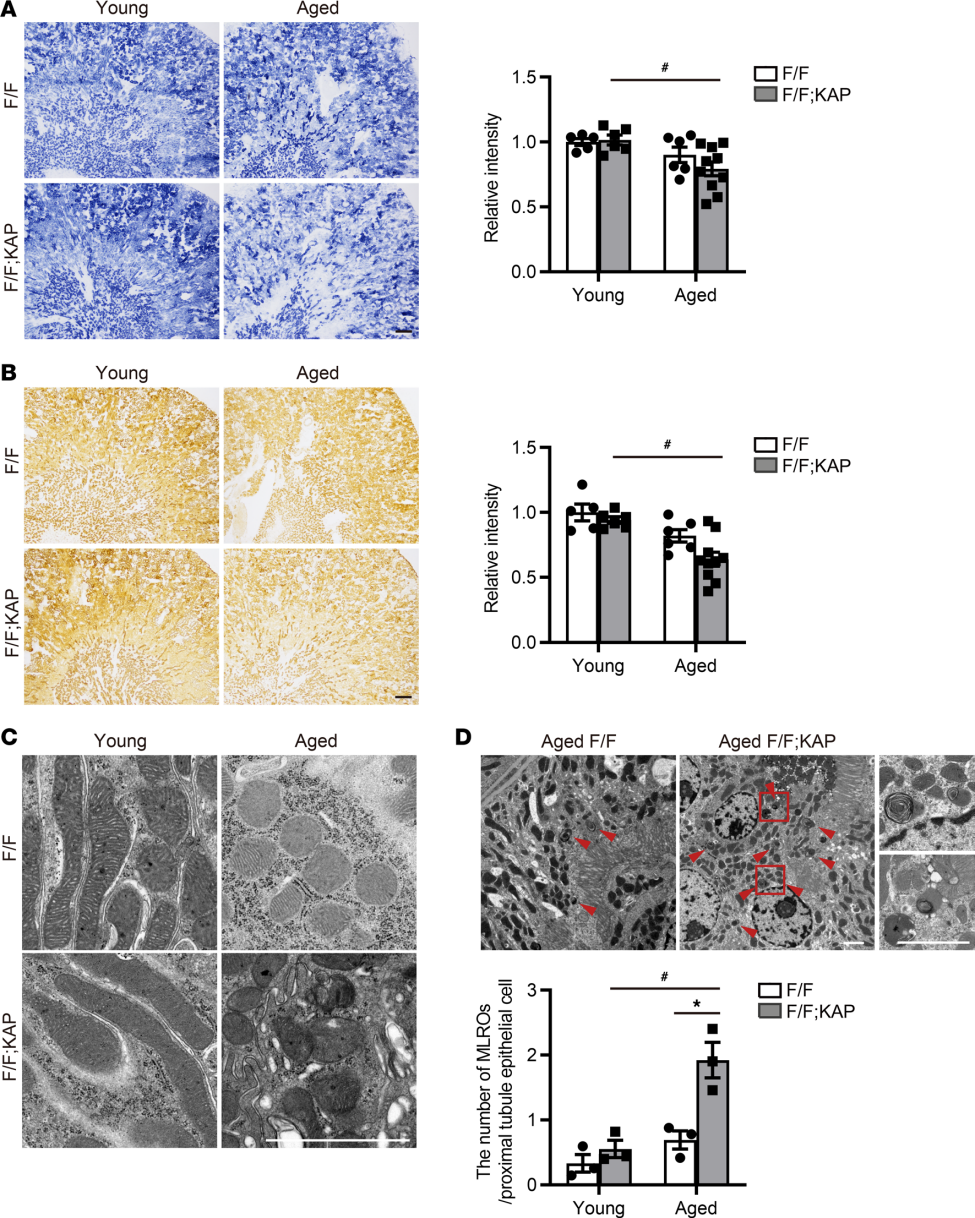
TFEB deficiency aggravates mitochondrial clearance in the S3 segment with age. (**A** and **B**) Representative images of succinate dehydrogenase (SDH) (**A**) and cytochrome c oxidase (COX) (**B**) staining in the kidneys of young and aged *Tfeb^fl/fl^* or *Tfeb^fl/fl^* KAP mice (*n* = 5–10). The relative staining intensity in the outer stripe of the outer medulla, which corresponds to the S3 segment, is shown. Values are normalized by the mean value of young *Tfeb^fl/fl^* mice. (**C** and **D**) Representative electron micrographs of the proximal tubules. Damaged mitochondria were observed in both aged *Tfeb^fl/fl^* mice and *Tfeb^fl/fl^* KAP mice (**C**). The number of mitochondria-lysosome–related organelles (MLROs) increased in aged *Tfeb^fl/fl^* KAP mice (**D**). Arrowheads and magnified images show MLROs. The number of MLROs was counted (*n* = 3). Scale bars: 250 μm (**A** and **B**) and 2 μm (**C** and **D**). Data are shown bar graphs, showing mean ± SEM. *P < 0.05 versus age-matched *Tfeb^fl/fl^* control littermates; ^#^*P* < 0.05 versus young mice (1-way ANOVA followed by the Tukey-Kramer test). F/F, *Tfeb^fl/fl^* mice; F/F;KAP, *Tfeb^fl/fl^* KAP mice.

On the basis of these scRNA-Seq analyses, we focused on mitochondria in the proximal tubules. Both the outer stripe of the outer medulla and the medullary ray of the cortex, which together correspond to the S3 segment, showed weak staining of succinate dehydrogenase (SDH) and cytochrome c oxidase (COX) in the kidneys of aged *Tfeb^fl/fl^* KAP mice (Figure 6, A and B). Next, we performed electron microscopy analysis to assess mitochondrial morphology. Damaged mitochondria were observed in both aged *Tfeb^fl/fl^* mice and aged *Tfeb^fl/fl^* KAP mice (Figure 6C). A recent study introduced an intracellular hybrid mitochondria–lysosome organelle, hereafter referred to as the mitochondria-lysosome–related organelle (MLRO), as an alternative mechanism for regulating mitochondrial homeostasis (55). That study showed that TFEB overexpression decreased the number of MLROs, likely via increased MLRO clearance, while blocking lysosomal degradation with leupeptin, a lysosome protease inhibitor, promoted MLRO accumulation. These findings indicate that lysosomal dysfunction due to TFEB downregulation may contribute to increased MLRO accumulation. Reflecting a downregulated lysosomal pathway, the number of MLROs in the proximal tubules was increased in aged *Tfeb^fl/fl^* KAP mice (Figure 6D). These results indicate that TFEB deficiency caused mitochondrial dysfunction by decreasing mitochondrial clearance in the S3 segment of aged mice.

### Age-related FAO dysregulation in the S3 segment likely affects systemic lipid metabolism.

**Figure 7:**
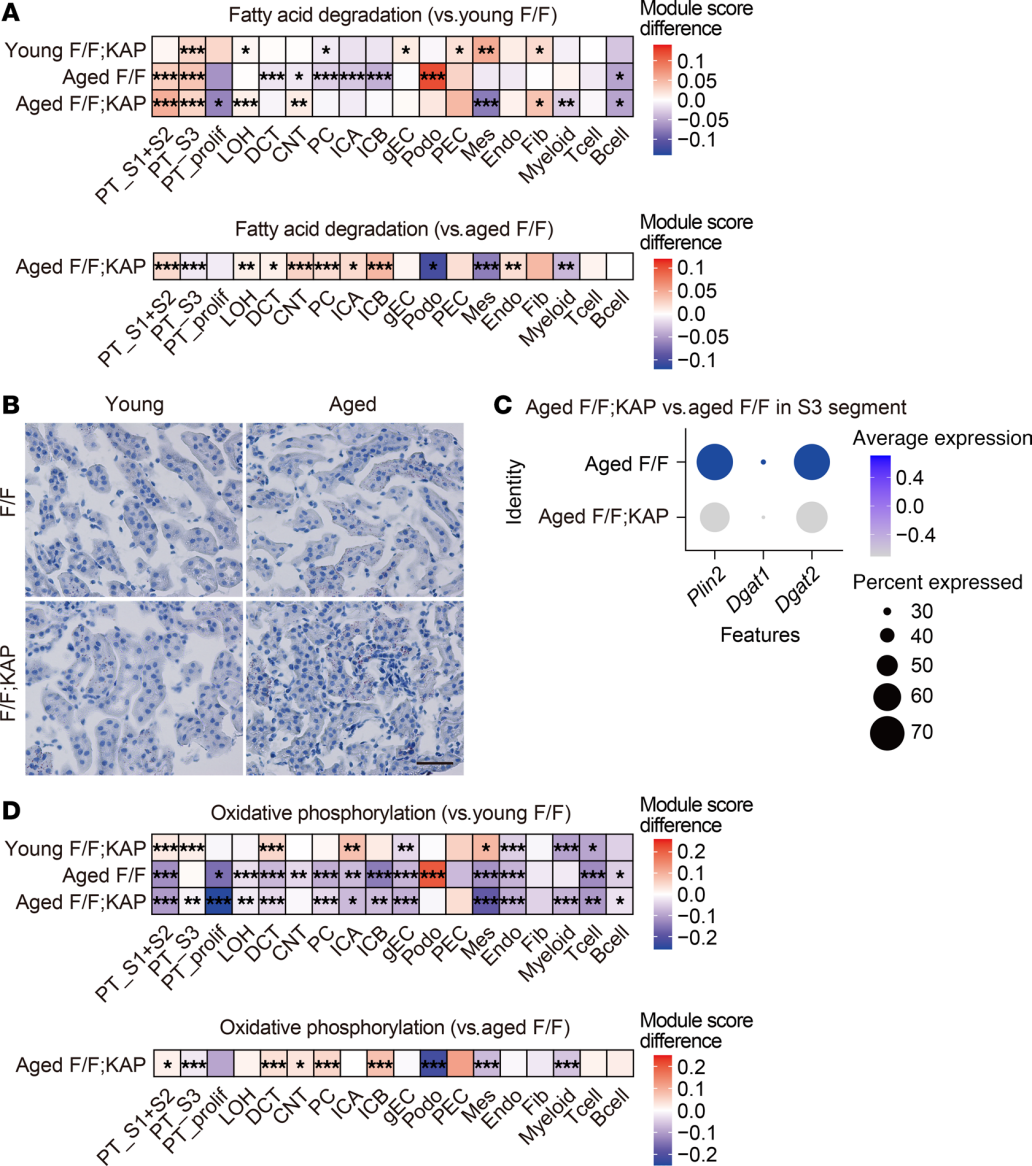
TFEB deficiency suppresses fatty acid oxidation in the S3 segment with age. (**A** and **D**) The module score of “Fatty acid degradation” and “Oxidative phosphorylation” across each cluster in the scRNA-Seq analyses. Upper heatmaps depict the difference between average scores of young *Tfeb^fl/fl^* KAP, aged *Tfeb^fl/fl^*, or aged *Tfeb^fl/fl^* KAP group and those of young *Tfeb^fl/fl^* group in each cluster. The module scores were compared in each cluster using 1-way ANOVA and then Dunnett’s test. Lower heatmaps depicting the difference between average scores of aged *Tfeb^fl/fl^* KAP group and those of aged *Tfeb^fl/fl^* group in each cluster. The module scores were compared in each cluster using 2-tailed Welch’s *t* test. *P < 0.05, **P < 0.01, and ***P < 0.001. (**B**) Representative images of Oil red O staining in the kidneys of young and aged *Tfeb^fl/fl^* or *Tfeb^fl/fl^* KAP mice (*n* = 5–10). Scale bars: 50 μm. (**C**) Dot plot of the scRNA-Seq analyses showing the expression of genes involved in LD formation in the S3 segment. Dot size denotes the percentage of cells expressing the marker genes. Color scale represents average gene expression values. F/F, *Tfeb^fl/fl^* mice; F/F;KAP, *Tfeb^fl/fl^* KAP mice.

FAO mainly occurs in mitochondria and involves the conversion of fatty acids (FAs) to acetyl-CoA, which can enter the TCA cycle. PTECs have a demand for high adenosine triphosphate (ATP), mainly generated by FAO and subsequent OXPHOS (56). We determined an FA degradation score for each cluster that was defined according to the expression of the KEGG gene list in the scRNA-Seq analyses. We observed higher expressions of FA degradation genes in the proximal tubule clusters of aged mice than in those of young *Tfeb^fl/fl^* mice (Figure 7A, Supplemental Figure 7A, and Supplemental Table 6). This indicates that aging may increase FAO dependency in the proximal tubules. However, the expression of FA degradation genes in the S3 segment cluster was lower in aged *Tfeb^fl/fl^* KAP mice than in aged *Tfeb^fl/fl^* mice (Figure 7A and Supplemental Table 7). In addition, compared with control HK-2 cells, those with TFEB knockdown showed a reduced difference in maximal OCR in the presence or absence of etomoxir, a carnitine palmitoyltransferase 1 (CPT1) inhibitor (Figure 5C). Considering the increased dependence on TFEB during kidney aging, these results indicate that TFEB deficiency suppresses the FAO activity in the proximal tubules of aged mice. Impaired FAO activity in the liver is associated with higher levels of circulating FFAs (54, 57). This suggests that suppressed FAO in the S3 segment may be related to increased plasma levels of circulating FFAs in aged *Tfeb^fl/fl^* KAP mice (Figure 3E). Despite the finding that LDs prevent lipotoxicity by FA sequestration (58, 59), little accumulation of renal LDs was found in aged *Tfeb^fl/fl^* KAP mice (Figure 7B). This observation may be caused by the downregulation of genes involved in LD formation (Figure 7C).

Contrary to the expression of FA degradation genes in the S3 segment cluster, in renal tubule clusters other than the S3 segment, such as the S1/S2 segments, the expression was higher in aged *Tfeb^fl/fl^* KAP mice than in aged *Tfeb^fl/fl^* mice (Figure 7A and Supplemental Table 7). In addition, KEGG pathway enrichment analyses and a score based on the expression of OXPHOS gene sets showed that the OXPHOS pathway was suppressed in the S3 segment cluster in aged *Tfeb^fl/fl^* KAP mice (Figure 5B) but was activated in other renal tubule clusters in these mice (Figure 7D, Supplemental Figure 7B, Supplemental Figure 8, and Supplemental Tables 8 and 9). Considering that fat accumulation in the liver and eWAT was increased in aged *Tfeb^fl/fl^* KAP mice (Figure 3, A, B, and D), these results indicate that the increase in circulating FFAs accompanied with FAO dysregulation in the S3 segment likely affects systemic lipid metabolism.

As for FA degradation and OXPHOS in other clusters, the scores in the podocyte, mesangial cell, and myeloid cell clusters of aged *Tfeb^fl/fl^* KAP mice were lower than those of aged *Tfeb^fl/fl^* mice (Figure 7, A and D). Given that a high-fat diet (FA-rich diet) damages these clusters (60, 61), the elevated plasma levels of FFAs in aged *Tfeb^fl/fl^* KAP mice may impair these clusters, while sparing the renal tubule clusters. Conversely, the FA degradation score in the endothelial cell cluster was increased (Figure 7A). This suggests that the suppression of the atherosclerosis pathway, induced by APOA4, may contribute to the improvement of FAO in this cluster of aged *Tfeb^fl/fl^* KAP mice (Supplemental Figure 8) (62, 63).

### Mitochondrial long-chain FAO is suppressed in the S3 segment of aged Tfeb^fl/fl^ KAP mice, but peroxisomal FAO compensates.

**Figure 8:**
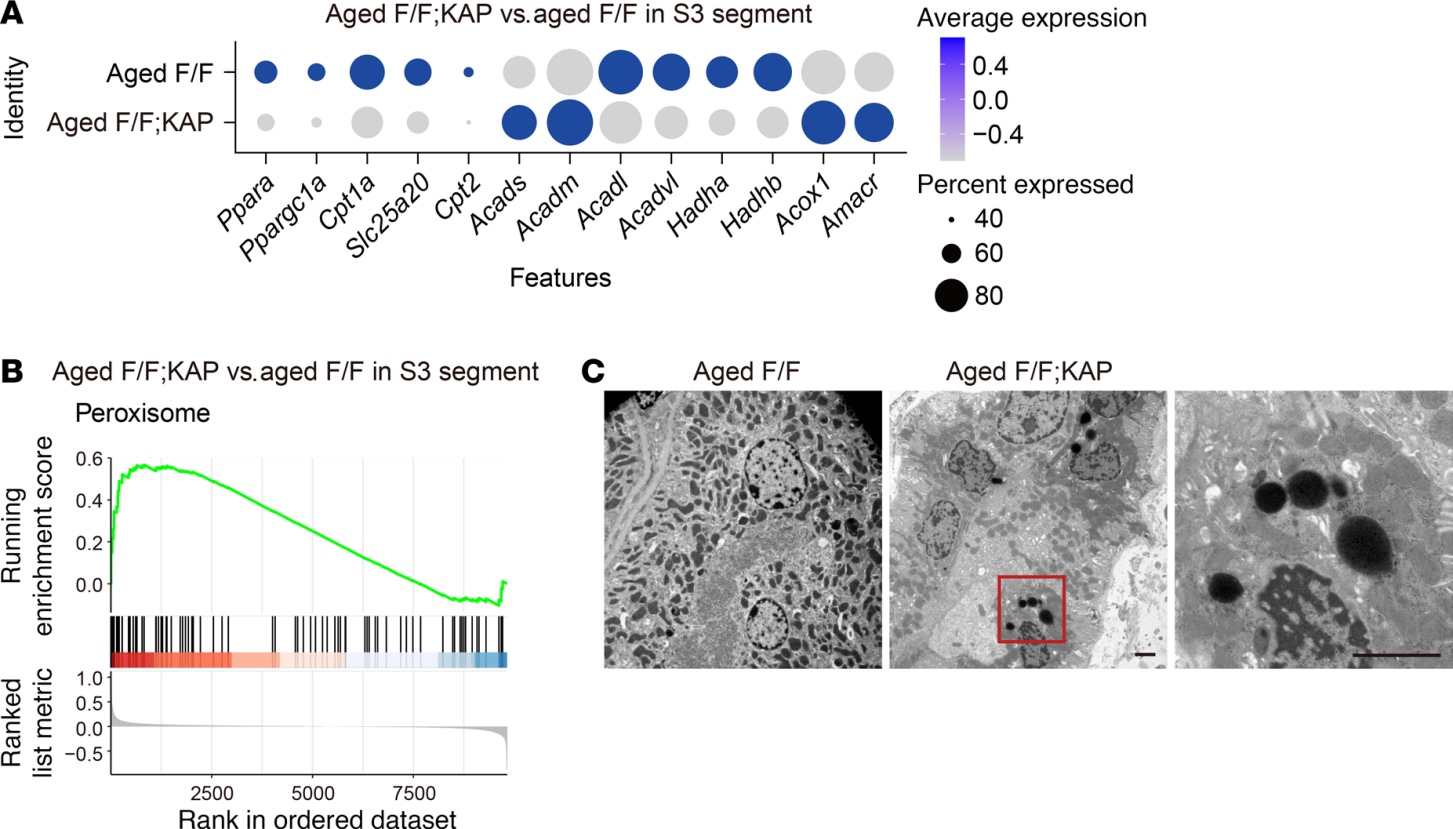
Peroxisomal FAO compensates for dysregulated mitochondrial FAO in the S3 segment with age. (**A**) Dot plot of the scRNA-Seq analyses showing the expression of genes involved in fatty acid oxidation in the S3 segment. Dot size denotes the percentage of cells expressing the marker genes. Color scale represents average gene expression values. (**B**) GSEA-based KEGG-enrichment plots show that the “Peroxisome” pathway in S3 segment was upregulated in aged *Tfeb^fl/fl^* KAP mice. The running enrichment score is plotted as a function of the position in the ranked list of genes. (**C**) Representative electron micrographs of the proximal tubules. Magnified images show peroxisomes. F/F, *Tfeb^fl/fl^* mice; F/F;KAP, *Tfeb^fl/fl^* KAP mice. Scale bars: 2 μm.

scRNA-Seq analyses further showed that genes involved in mitochondrial long-chain FAO, including *Hadha* and *Hadhb*, were downregulated in the S3 segment cluster of aged *Tfeb^fl/fl^* KAP mice, whereas genes involved in peroxisomal FAO, including *Acox1* and *Amacr,* were upregulated (Figure 5A and Figure 8A). Peroxisomal FAO has been shown to compensate for reduced mitochondrial FAO (64, 65). The peroxisome pathway was indeed activated in the S3 segment cluster of aged *Tfeb^fl/fl^* KAP mice (Figure 5B and Figure 8B). Electron microscopy analysis also showed the proliferation of peroxisomes (Figure 8C). These results indicate that peroxisomal FAO compensates for dysregulated mitochondrial FAO in the S3 segment of aged *Tfeb^fl/fl^* KAP mice.

## Discussion

**Figure 9:**
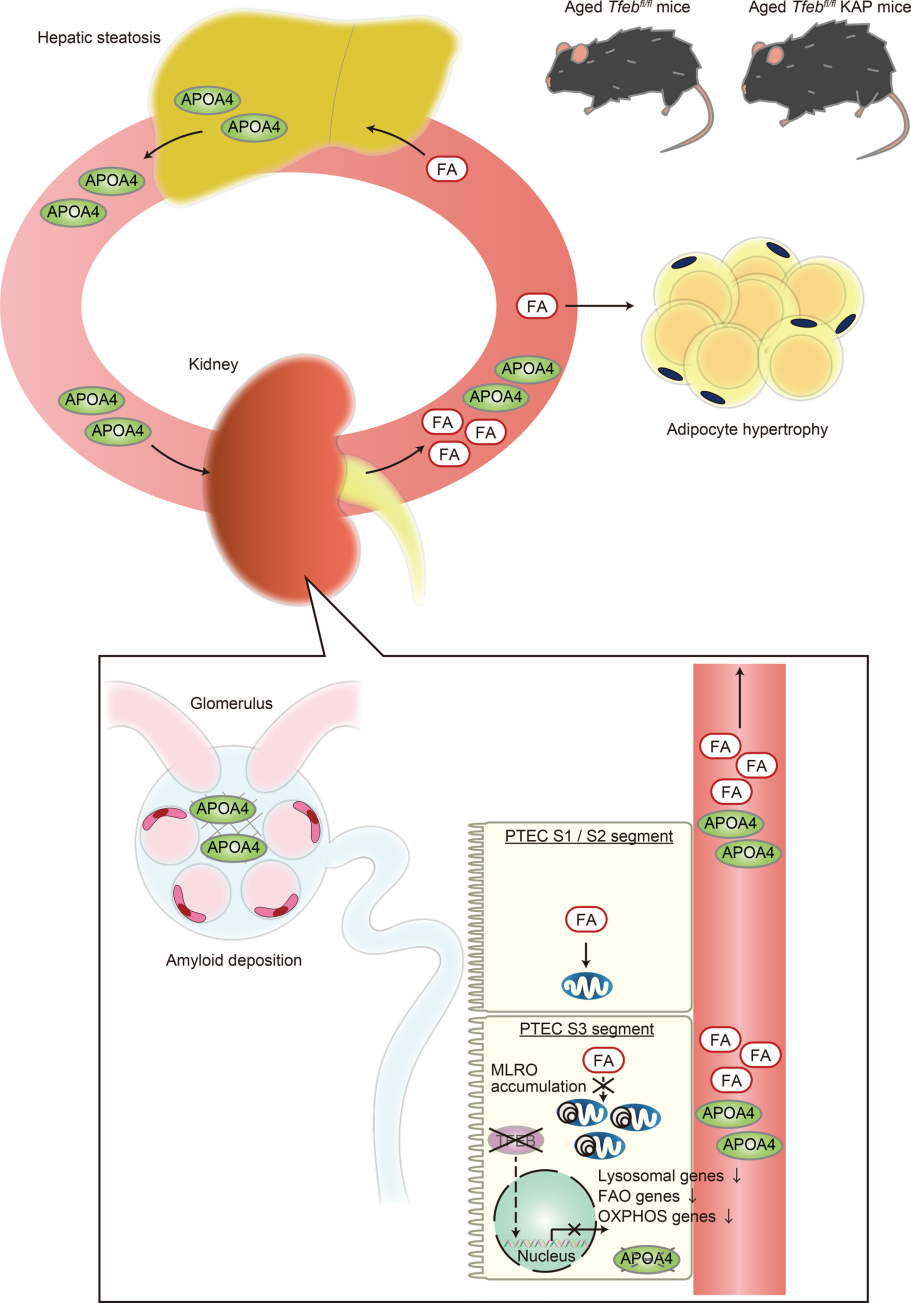
TFEB deficiency in the S3 segment of the proximal tubules causes systemic metabolic change and occasionally leads to APOA4 amyloidosis with age. Schematic illustration of this study. TFEB deficiency in the S3 segment of the proximal tubule causes mitochondrial dysfunction in aged mice due to the following reasons: (a) MLRO accumulation due to downregulation of lysosomal pathway, indicative of decreased mitochondrial clearance, and (b) downregulation of both fatty acid oxidation (FAO) pathway and oxidative phosphorylation (OXPHOS) pathway. TFEB deficiency also causes increased circulating free fatty acid (FA), hepatic steatosis, and adipocyte hypertrophy. Both increased hepatic APOA4 synthesis due to hepatic steatosis and decreased APOA4 degradation in TFEB-deficient PTECs may cause APOA4 amyloidosis.

There were 3 main findings in this study (Figure 9). First, nuclear TFEB localization in PTECs decreased with age in both mice and patients with CKD. TFEB deficiency in PTECs caused mitochondrial dysfunction and increased circulating FFAs, which can lead to systemic metabolic disorders such as hepatic steatosis and adipocyte hypertrophy. Second, both increased hepatic APOA4 synthesis due to hepatic steatosis and decreased APOA4 degradation in TFEB-deficient PTECs are likely the presumptive mechanism of APOA4 amyloidosis. Third, scRNA-Seq analysis and electron microscopy revealed that the mitochondrial dysfunction in PTECs was caused by transcriptional downregulation of the FAO and OXPHOS pathways, as well as by MLRO accumulation, which results from downregulation of the lysosomal pathway and is indicative of decreased mitochondrial clearance. These results provide fundamental insights into the presumptive mechanisms of APOA4 amyloidosis and dysregulated systemic metabolism in elderly patients with CKD.

Nuclear TFEB localization in PTECs decreased with age in this study, but TFE3 localization remained unchanged. In aged mice, TFEB deficiency downregulated TFEB downstream signaling pathways such as the lysosomal pathway and the FAO pathway, whereas it did not do so in young mice. TFE3 can compensate for chronic TFEB deficiency (66), but this may occur only in young mice and not in aged ones, the latter of which have a higher demand for TFEB/TFE3, although nuclear TFE3 localization remains infrequent regardless of age. This indicates that TFEB plays an important role in kidney aging.

We demonstrated that TFEB deficiency in PTECs may cause APOA4 amyloidosis in aged mice. LMD–LC-MS/MS analysis showed that APOA2 exhibited the second highest spectral count among amyloidogenic proteins. Recent studies reported that proteome profiles of amyloid deposits in individuals with APOA4 amyloidosis demonstrated the presence of APOA2 and vice versa (45, 47, 67). This suggests that APOA4 and APOA2 are inextricably linked and that differentiating APOA4 amyloidosis from APOA2 amyloidosis is difficult. However, the results of LMD–LC-MS/MS analysis and the tissue distribution of amyloid deposition are similar to those previously reported in APOA4 amyloidosis (47), and we conclude that APOA4 amyloidosis was the most likely type. SAA protein, the amyloidogenic protein responsible for amyloid A (AA) amyloidosis, also exhibited the second highest spectral count in this study. Aged WT mice are known to occasionally develop AA amyloidosis (68), and some aged *Tfeb^fl/fl^* mice also showed amyloid deposits, albeit to a lesser extent than *Tfeb^fl/fl^* KAP mice. Therefore, it is possible that aged *Tfeb^fl/fl^* KAP mice may codevelop AA amyloidosis.

Various factors are involved in enhancing amyloid formation, including aging, high concentrations of amyloidogenic proteins, and genetic mutations. Aging can be a major risk factor for amyloid deposition even in the absence of genetic mutations or large increases in concentration of amyloidogenic proteins (69, 70). This influence of aging may be attributed to the inflammatory environment generated by senescence-associated secretory phenotype (SASP) factors (67). In terms of concentration, we found that APOA4 regulation depends on TFEB in PTECs. APOA4 is a 46 kDa apolipoprotein that circulates as a monomer or a dimer and is partially associated with high-density lipoproteins (HDLs) (71, 72). A fraction of APOA4, including monomers that have a relatively smaller molecular weight and are often present in HDL-unbound states, is filtered through the glomeruli, followed by reabsorption and degradation in the proximal and distal tubules (50, 73). However, the mechanism of reabsorption and degradation in the proximal tubules is largely unknown. This study shows that TFEB deficiency increased APOA4 accumulation in PTECs, suggesting that lysosomal function in the proximal tubules is important because, in rats, APOA4 in the kidney is increased by leupeptin, a lysosomal protease inhibitor (49), and TFEB is the master regulator of the lysosomal pathway (21). In addition, there have been recent reports of an APOA4 signal sequence that may be involved in amyloid seeds and pathogenic APOA4 variants that are predicted to expand the amyloidogenic hotspot (74, 75). Considering the results of in vivo and human autopsy samples, the age-related decrease in nuclear TFEB localization of PTECs may induce APOA4 amyloidosis in aged patients. However, unlike aged *Tfeb^fl/fl^* KAP mice, TFEB leaves some residual activity in elderly humans, which may contribute to the lower frequency of APOA4 amyloidosis in humans. Rather, genetic variants expanding the amyloidogenic hotspot may play a more crucial role in human APOA4 amyloidosis. As described above, the causes of APOA4 amyloidosis are diverse, and further elucidation of the pathogenesis of APOA4 amyloidosis is expected in the future.

The clinical characteristics of patients with APOA4 amyloidosis include a gradual decline in renal function, infrequent proteinuria, and diagnosis in old age, and the medulla is the primary site of involvement (76). These features reduce the chances of renal biopsy, resulting in missed diagnoses. In fact, there may be many patients with unidentified APOA4 amyloidosis, considering the findings of elevated plasma APOA4 concentrations in patients with CKD (75, 77). Since nuclear TFEB localization in PTECs decreases with age and renal dysfunction, and the prevalence of hepatic steatosis is high in patients with CKD (20, 78), decreased nuclear TFEB localization in PTECs may lead to APOA4 amyloidosis in the elderly due to increased hepatic APOA4 synthesis and decreased APOA4 degradation, as in aged *Tfeb^fl/fl^* KAP mice. However, phenotypic differences between animal species should be noted. In patients with APOA4 amyloidosis, amyloid deposits are considered to be restricted to the renal medulla and to be absent in the glomeruli, interstitium, and vessels of the renal cortex (76). On the other hand, both the present study and previous reports showed that, in mice with APOA4 amyloidosis, amyloid fibrils were prominently deposited in the glomeruli (47, 67). Furthermore, APOA4 amyloidosis that was reported in a species of monkey, the Cotton-top tamarin, also exhibited obvious glomerular deposition (79). In addition to differences between species, genetic mutations may contribute to phenotypic diversity. The patient with APOA4 amyloidosis, whose sample was used in this study and whose *APOA4* sequence variants differ from those of other reports, showed amyloid deposition in the glomeruli and vessels of the renal cortex (Figure 2F) (48, 75). The peptides identified in aged *Tfeb^fl/fl^* KAP mice and this patient with APOA4 amyloidosis did not contain a signal sequence involved in amyloid seeds (74). However, we identified similar peptide regions in aged *Tfeb^fl/fl^* KAP mice, the patient with APOA4 amyloidosis showing glomerular amyloid deposition, and Cotton-top tamarin with APOA4 amyloidosis (Supplemental Figure 9). Any of these peptides could be signal sequences involved in APOA4 amyloidosis with glomerular deposition, and further studies on phenotypic differences are needed. Additionally, the glomeruli in aged *Tfeb^fl/fl^* KAP mice may provide an inflammatory microenvironment that serves as a scaffold for amyloid deposition, as indicated by lower FA degradation and OXPHOS scores in the podocyte, mesangial cell, and myeloid cell clusters (Figure 7, A and D).

By confirming the presence of APOA4 amyloidosis, we demonstrated that the loss of TFEB in PTECs caused metabolic disorders in aged mice. Given that nuclear TFEB localization declines with age or decreased renal function (20), TFEB deficiency in PTECs is considered to be one of the reasons for dyslipidemia in patients with CKD (80). We speculate that the metabolic disorders in aged *Tfeb^fl/fl^* KAP mice may be caused by several factors. The first of these is increased food intake that may cause the elevation of plasma FFA levels. Since dietary fat absorption results in increased intestinal APOA4 synthesis in a dose-dependent manner (81), the lack of change in intestinal *Apoa4* mRNA levels in aged *Tfeb^fl/fl^* KAP mice indicates that the elevated plasma FFA levels are unlikely to be related to increased caloric intake (Supplemental Figure 4D). Next, the origins of FFAs must be considered. When circulating FFAs are derived from adipose tissue, as in fasting or insulin-resistant conditions, the proximal tubules uptake FFAs and form LDs (15, 82). Plasma insulin was not elevated in aged *Tfeb^fl/fl^* KAP mice (Supplemental Figure 5D), indicating that insulin resistance likely has no effect on circulating FFAs. In addition, the expression of adipogenic genes and the gene that participates in LD formation increased in eWAT of aged *Tfeb^fl/fl^* KAP mice. On the other hand, there was little accumulation of renal LDs in aged *Tfeb^fl/fl^* KAP mice. Based on these results, the elevated plasma FFA levels may be related to unprocessed FAs due to mitochondrial dysfunction with impaired FAO activity in PTECs. scRNA-Seq analyses in this study showed that TFEB deficiency in the S3 segment downregulated both the FAO and OXPHOS pathways in aged mice. Additionally, the S3 segment may be highly dependent on FAO in aged mice, considering that the FA degradation pathway, whose genes were most highly expressed in this segment, was further activated with age, while the OXPHOS pathway was not suppressed (Figure 4D, Supplemental Figure 6C, and Supplemental Figure 7A). The downregulation of FAO pathways by TFEB deficiency and dependency on FAO indicates that the metabolic disturbances in aged *Tfeb^fl/fl^* KAP mice may be associated with the specificity of the S3 segment. Further research is needed to examine the mechanism of the elevated plasma FFA levels and whether the metabolic disorders are also caused by the deficiency of TFEB in other segments, such as the S1/S2 segments. Furthermore, we demonstrated in addition to transcriptional alterations, MLRO accumulation led to mitochondrial dysfunction in aged *Tfeb^fl/fl^* KAP mice. MLRO accumulation is negatively regulated by lysosomal function and is associated with cellular dedifferentiation (55). Given that the number of dedifferentiated PTECs increases with age (83), MLRO accumulation during the dedifferentiation process in aging may be exacerbated by TFEB deficiency.

Long-term loss of TFEB in PTECs did not significantly affect renal morphologic features or renal function, except for causing amyloidosis. We speculate that the efferocytosis of senescent cells and compensation by peroxisomal FAO are responsible for this phenotype. First, senescent epithelial cells, which consist predominantly of PTECs and distal tubular epithelial cells in the aging kidney (84), escape from efferocytosis (85) and secrete SASP factors, leading to chronic inflammation and renal dysfunction (86). Since TFEB is required for the survival of senescent cells (87), it is possible that senescent cells in the S3 segment of aged *Tfeb^fl/fl^* KAP mice were removed as a result of TFEB deficiency, preventing the worsening of renal function (Supplemental Figure 3C). This is reflected by activation of the efferocytosis pathway and a decreased proportion of the S3 segment cluster (Figure 5B and Supplemental Figure 6G). Second, it was recently reported that tubule-specific deletion of CPT1A, the rate-limiting enzyme for long-chain FAO, had no significant effect on kidney function in mice due to compensation by peroxisomal FAO after aging (65). In the S3 segment cluster of aged *Tfeb^fl/fl^* KAP mice, genes involved in peroxisomal FAO and mitochondrial middle- or short-chain FAO, including *Acadm* and *Acads*, were upregulated, whereas genes involved in mitochondrial long-chain FAO were downregulated (Figure 8A). This is considered to have led both to oxidation of long-chain FAO, which is compensated for by peroxisomes, and to subsequent further metabolism in the mitochondria.

There are some limitations in this study. Since we did not expect amyloidosis to be closely related to metabolism, we did not measure daily food intake or fasting plasma concentrations, and we did not perform indirect calorimetry, insulin tolerance tests, or glucose tolerance tests. Thus, we cannot completely exclude the possibility that metabolic disorders are associated with energy expenditure, dietary intake, or insulin resistance. More importantly, changes in mRNA abundance do not necessarily correlate with FAO activity, and the metabolic disorders are caused by both age-specific and S3 segment–specific factors. Therefore, to verify the mechanism by which tubular TFEB deficiency in aging causes elevated plasma FFA levels, future studies should use advanced technologies such as spatial metabolomics in combination with isotope tracing and matrix-assisted laser desorption/ionization MS imaging (88).

In summary, we report that TFEB deficiency in the S3 segment of the proximal tubules of aged mice caused metabolic disorders and occasionally led to APOA4 amyloidosis. These results reveal a presumptive mechanism for the pathogenesis of APOA4 amyloidosis and suggest that TFEB activators, including trehalose (35, 89), may be a therapeutic strategy for CKD-related metabolic disorders and age-related APOA4 amyloidosis.

## Methods

### Sex as a biological variable.

Our study examined male mice because KAP promoter is androgen inducible.

### Mice.

PTEC-specific *Tfeb*-overexpressing mice on a C57BL/6N background were generated by crossing *Tfeb*^fs/fs^ transgenic mice (22) with KAP-Cre mice (gifted by T. Matsusaka and F. Niimura, Tokai University School of Medicine, Kanagawa, Japan) (11). *Tfeb^fl/fl^* KAP mice have been described previously (36). All male mice had ad libitum access to water and diet. They were housed in box cages and maintained on a 12-hour light/12-hour dark cycle.

### Biochemical measurements.

Blood samples and urine samples were collected from mice under anesthesia. Plasma was obtained after centrifugation (15 minutes, 845*g*, 4°C) and concentrations of cystatin C, glucose, total cholesterol, triglycerides, nonesterified fatty acids (NEFA), phospholipid, and insulin were measured using the Mouse/Rat Cystatin C Quantikine ELISA Kit (R&D Systems, MSCTC0), Mouse APOA4 ELISA Kit (Abbexa, abx258379), the Cholesterol E-test (Wako, 439–17501), the Triglyceride E-test (Wako, 432–40201), the NEFA C-test (Wako, 279–75401), the Phospholipid C-test (Wako, 433–36201), and Mouse/Rat Insulin ELISA kit (Morinaga, M1108). Urine samples were obtained after centrifugation (10 minutes, 1,000*g*, 4°C), and concentrations of albumin and creatinine were measured using the Mouse Albumin ELISA Kit (Bethyl, E99-134) and QuantiChrom Creatinine Assay Kit (BioAssay Systems, DICT-500). All kits were used in accordance with the manufacturer’s protocols.

### Single-cell isolation of the kidney and sequencing.

Kidneys obtained from young or aged *Tfeb^fl/fl^* mice and *Tfeb^fl/fl^* KAP mice were subjected to scRNA-Seq analyses. All mice were perfused with phosphate-buffered saline (PBS) via the left ventricle to remove blood cells. Afterward, the kidneys were harvested, and the renal cortex was dissected using a razor blade after the renal capsule was removed. Renal cortex samples from 2 or 3 mice in each group were combined. These samples were minced with a sterile razor blade and digested in 3.0 mL of HBSS (Thermo Fisher Scientific, 14025092) containing 250 μg/mL Riberase TH (Roche, 5401151001) and 100 U/mL DNase I (Roche, 11284932001) for 30 minutes at 37°C with shaking at 200 rpm. After digestion, the cell suspension was centrifuged at 200*g* for 4 minutes. After removing the supernatant, pellets were washed twice with 2.0% bovine serum albumin (BSA) (Sigma-Aldrich, A3059) in PBS. Finally, the cell suspension was filtered through a 40 μm cell strainer (Falcon, 352340), and the cells were collected by centrifugation (4 minutes, 200*g*) and were resuspended with 2.0% BSA in PBS. Cell viability was investigated by trypan blue staining, and cell concentration was assessed using a hemocytometer. Libraries of the single cells were prepared using Chromium Next GEM Single Cell 3’ Library and Gel Bead Kit v3.1 according to the manufacturer’s instructions (10× Genomics). They were sequenced on a DNBSEQ-G400 (MGI Tech).

### scRNA-Seq data processing, normalization, integration, and cell population identification.

The 10× Chromium raw sequencing data were first processed with Cell Ranger (version 6.0.0). SoupX (version 1.6.2) was used to determine ambient RNA contamination and was then applied for background correction (90). Seurat (version 4.4.0) package was then applied (91). Initially, genes expressed in more than 3 cells and cells with at least 200 genes were retained. Cells with fewer than 1,000 feature counts and more than 50% mitochondrial counts were further filtered. Gene expression levels for each cell were normalized by the total expression, multiplied by 10,000, and then log-transformed. The top 2,000 highly variable genes (HVGs) were identified using the variance-stabilizing transformation (VST) method and were scaled based on their average expression and dispersion. The scaled data were then subjected to principal component analysis (PCA) using HVGs and the dimension was reduced. In addition, we identified doublets using DoubletFinder (version 2.0.3) and excluded cells identified as doublets (92). Then, we used Harmony (version 1.0.3) using RunHarmony function to integrate datasets with batch effect correction (93). As a result, 30,145 cells were included in downstream analyses. To cluster the cells, Louvain algorithm was applied based on the first 30 PCs using FindNeighbors and FindClusters. Uniform Manifold Approximation and Projection (UMAP) was used for visualization. To annotate the clusters, cluster biomarkers were identified by FindAllMarkers with the Wilcoxon rank-sum test.

### Differentially expressed genes (DEGs) analysis and KEGG pathway gene set enrichment analysis.

DEGs between young or aged *Tfeb^fl/fl^* mice and *Tfeb^fl/fl^* KAP mice in each cluster were identified using model-based analysis of single-cell transcriptomics (MAST), which was implemented in the FindMarkers using the following parameters: log_2_ fold change threshold = 0.20, minimum percentage of cells expressing the genes = 0.05, and adjusted *P* < 0.05 (94). KEGG pathway gene set enrichment analyses (GSEA) were conducted using the gseKEGG in the clusterProfiler package (version 4.6.2) (95).

### FA degradation and OXPHOS scoring.

To evaluate FA degradation and OXPHOS across clusters, we produced gene sets involved in “Fatty acid degradation (mmu00071)” and “Oxidative phosphorylation (mmu00190)” from KEGG PATHWAY Database at GenomeNet (https://www.genome.jp/kegg/pathway.html) (Supplemental Table 10). FA degradation and OXPHOS scores were evaluated using AddModuleScore function implemented in Seurat with default parameter. To evaluate these scores in each cluster, we calculated the average module scores across each cluster and compared the scores of young *Tfeb^fl/fl^* KAP, aged *Tfeb^fl/fl^*, or aged *Tfeb^fl/fl^* KAP group with those of young *Tfeb^fl/fl^* group using 1-way ANOVA and then Dunnett’s test. The module scores of aged *Tfeb^fl/fl^* KAP group were also compared with those of aged *Tfeb^fl/fl^* group in each cluster using 2-tailed Welch’s *t* test.

### Cell culture.

HK-2 cells (human PTECs) were obtained from ATCC (CRL-2190). HK-2 cells were grown in DMEM/F-12 (Thermo Fisher Scientific, 11320033) with 10% FBS and 1% penicillin/streptomycin (Sigma-Aldrich, P4333). HK-2 cells were treated using recombinant human APOA4 protein with His-Tag (Sino Biological, 16082-H08H) for 3 hours. For TFEB knockdown, HK-2 cells were transfected with ON-TARGETplus Human TFEB siRNA (Dharmacon, L-009798-00-0005) using Lipofectamine RNAiMAX Transfection Reagent (Thermo Fisher Scientific, 13778150) and the transfected cells were used for the subsequent experiment after 48 hours. HK-2 cells were transfected with ON-TARGETplus Nontargeting Control Pool (Dharmacon, D-001810-10-05) as a control. The silencing efficiency was assessed by Western blotting.

### Antibodies.

The antibodies used are listed in Supplemental Table 11.

### Histological analysis.

Histological analysis was performed as described previously, with modifications (16). The following were also performed as described previously: antigen retrieval on paraffin-embedded sections; electron microscopy analysis; PAS staining; H&E staining; Oil red O (ORO) staining; and COX/SDH staining (14, 15, 39, 96). Assessment of kidney injury was performed (14, 16, 20, 96). The size of adipocyte and ORO^+^ LD was measured using ImageJ. For COX/SDH staining, high-power fields in the outer stripe of the outer medulla, which correspond to the S3 segment, were captured and quantified using ImageJ. Picrosirius red staining and Congo red staining were performed by the manufacturer (Applied Medical Research Laboratory). The degree of the amyloid deposition was scored from 0 to 3 (0, absent; 1, multifocal minimal; 2, mild to moderate; 3, extensive), according to the previous reports, with modifications (47). For assessment of TFEB localization, we performed immunofluorescence staining using fixed-frozen tissue sections (tissues were fixed with 4% paraformaldehyde in PBS for 6 hours, cryoprotected with 30% sucrose in PBS for 2 days, and then embedded in optimal cutting temperature compound; Sakura Finetek, 4583). These tissue sections were harvested under ad libitum feeding. Nuclear TFEB localization was evaluated by the percentage of PTECs exhibiting nuclear TFEB staining, as described previously (35). In all quantitative or semiquantitative analysis of histological staining, at least 10 high-power fields (for the size of ORO^+^ LD, COX/SDH staining, and nuclear TFEB staining) or low-power fields (for the size of adipocyte and Picrosirius red staining) in each kidney were reviewed by 2 nephrologists in a blinded manner. For electron microscopy analysis, fibril diameters were measured using ImageJ, and the number of MLROs in PTECs was counted. MLROs are defined as electron-dense ‘‘lysosome-like’’ structures approximately 0.5–1 μm in diameter, which appeared to be either a single or double membrane bound and contained undegraded electron-dense onion-like membranes with other heterogeneous content (55). MLROs are morphologically distinct from the mitophagosome, which are defined as double-membraned autophagosomes enveloping mitochondria and single-membraned late autolysosomes containing degraded mitochondria. At least 20 PTECs in each kidney were analyzed by 2 nephrologists in a blinded manner.

### Tissue microdissection.

Sections of FFPE samples (10 μm thick) were placed on polyethylene napthalate (PEN) membrane slides (Leica Microsystems, 11505158) precoated with poly-L-lysine (Sigma-Aldrich, P8920) for LMD. Sections were air dried, melted, and deparaffinized, and H&E staining was performed. Glomeruli with amyloid deposition were microdissected into 0.5 mL tube caps (Corning, PCR-05-C) containing 35 μL 10 mM Tris /1mM EDTA/0.002% ZWITTERGENT 3-16 (Santa Cruz Biotechnology Inc., sc-281194A) buffer using the LMD7000 (Leica Microsystems). Collected sample was heated at 98°C for 90 minutes with occasional vortexing. Following 30 minutes of sonication in a waterbath, samples were reduced with TCEP-HCl (Thermo Fisher Scientific, 20490), alkylated with iodoacetamide (Wako, 099-05591), and digested with Trypsin/Lys-C Mix (Promega, V5073) at 37°C overnight.

### LC-MS/MS analysis.

LC-MS/MS analysis of the digested peptides was performed using UltiMate 3000 Nano LC systems (Thermo Fisher Scientific) interfaced with a mass spectrometer (Q-Exactive, Thermo Fisher Scientific). The digested peptides were separated using an 3 μm C18 NANO HPLC CAPILLARY COLUMN100-3-12 (NTCC-360/100-3-125, 125 × 0.1 nm, Nikkyo Technos) and eluted with a linear gradient of 5%–90% buffer B (acetonitrile containing 0.1% formic acid) in buffer A (water containing 0.1% formic acid) at a flow rate of 300 nL/min. Data were acquired using the data-dependent analysis mode. The resulting data were analyzed with Mascot Di stiller v2.5, Mascot Server v2.5 (Matrix Science), and Scaffold software version 3.0 (Proteome Software Inc.). The resulting data were collated using a mouse database (taxonomy ID 10090) obtained from the UniProt database (http://www.uniprot.org/, last accessed February 5, 2017).

### Measurement of mitochondrial respiratory function.

The mitochondrial OCR was measured using Seahorse XF96 analyzer as described previously with slight modifications (97, 98). HK-2 cells were transfected with TFEB siRNA for 48 hours, followed by Seahorse XF DMEM (pH 7.4; Agilent Technologies, 103578-100) — supplemented with 10 mM glucose (Agilent Technologies, 103577-100), 1 mM pyruvate (Agilent Technologies, 103578-100), and 2 mM L-glutamine (Agilent Technologies, 103579-100) — with or without 40 μM etomoxir (Sigma-Aldrich, E1905) for 1 hour. The OCR was measured under the basal condition and in the presence of 1.5 μM oligomycin, 0.5 μM carbonyl cyanide 4-(trifluoromethoxy)phenylhydrazone (FCCP), and 0.5 μM rotenone/antimycin A using Seahorse XF Cell Mito Stress Test Kit (Agilent Technologies, 103015-100). The change in maximal (Δ maximal) OCR was determined by subtracting the maximal OCR in the etomoxir-treated group from the OCR in the etomoxir-untreated group. OCR was analyzed with Wave software v2.6.1 (Agilent Technologies).

### Quantitative PCR and Western blot analysis.

Quantitative PCR (qPCR) and Western blot analyses were performed as described previously (99). The sequences of the primers are listed in Supplemental Table 12.

### Human kidney specimens.

We analyzed human kidney specimens obtained from patients who had undergone renal biopsy at the Osaka University Hospital to assess nuclear TFEB localization, using the dataset previously reported in our papers (20, 35). These specimens were taken in the morning, from patients who skipped breakfast. Tissues were fixed with 10% neutral buffered formalin for 1 day and then embedded in paraffin. TFEB deficiency in PTECs leads to systemic metabolic changes in aged mice but not in young mice (Figure 3, A–D, and Supplemental Figure 5, A and B). Therefore, we included aged patients with obesity to avoid an overestimation of the true ratio of TFEB nuclear localization, while we excluded young patients with obesity with decreased TFEB activity in PTECs (35). In addition, we used human autopsy specimens at Osaka University Hospital and Sapporo Medical University Hospital to evaluate the relationship between proximal tubular nuclear TFEB localization and APOA4 amyloid deposition.

### Statistics.

In vivo results are presented as bar graphs, with data expressed as mean ± SEM, and results obtained from patients are presented as box plots showing median values and interquartile ranges. Statistical analyses were conducted using JMP Pro 17 software (JMP Statistical Discovery) and GraphPad Prism 8 (GraphPad Software). Multiple-group comparisons were performed using 1-way ANOVA with post hoc testing using the Tukey-Kramer test. In Figure 7, A and D, and Supplemental Table 6 and 8, 1-way ANOVA and then Dunnett’s test were used to detect intergroup differences. The difference between 2 experimental values was assessed using the 2-tailed Student’s *t* test, Pearson’s χ^2^ test (in supplemental tables 1 and 2), or Wilcoxon rank-sum test. Mixed-effects analysis was used to assess body weight progress. Statistical significance was defined as *P* < 0.05. The statistical analysis used for scRNA-Seq is shown in the methods related to scRNA-Seq.

### Study approval.

All animal experiments were approved by the Animal Research Committee of Osaka University and conformed to the Japanese Animal Protection and Management Law (No. 25). All human studies were approved by the IRB of Osaka University Hospital (nos. 17334, 20504, and 15234-8) and Sapporo Medical University Hospital (no. 312-214) and adhered to the Declaration of Istanbul. We have complied with all the relevant ethical regulations, and informed consent was obtained.

### Data availability.

The scRNA-Seq data set has been deposited in the Gene Expression Omnibus (GEO) repository (GSE270205). The proteomics raw data have been deposited to the ProteomeXchange Consortium via the jPOST partner repository (100) with the dataset identifier PXD055311. Any additional information required to reanalyze the data reported in this paper is available from the corresponding author upon request. All the raw data for graphs are provided in the Supporting Data Values file.

## Author contributions

JN, T Yamamoto, and YT designed the study; JN carried out most experiments, analyzed and interpreted the data, and drafted the manuscript; T Yamamoto interpreted data and edited/revised the manuscript; TNH, A Takahashi, JM, S Minami, SS, HY, S Maeda, S Matsui, and HK helped histologic analyses; T Yamamuro. and ST helped metabolic analyses; A Takasawa evaluated human autopsy specimens; RE and YO helped with scRNA-Seq analysis; IM, T Yoshimori, AB, and YI provided intellectual input; and all authors contributed to the discussions and approved the final version of the manuscript. The order of co–first authorship was decided on the absolute amount of time spent on the project.

## Supplementary Material

Supplemental data

Unedited blot and gel images

Supplemental tables 1-12

Supporting data values

## Figures and Tables

**Table 1:**
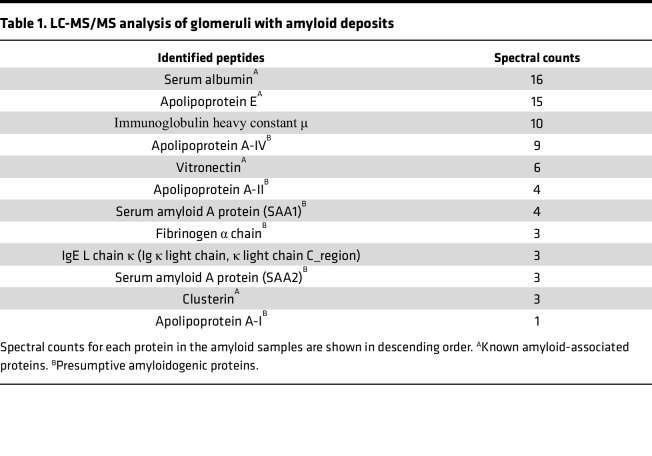
LC-MS/MS analysis of glomeruli with amyloid deposits

## Version 1 12/19/2024

In-Press Preview

## Version 2 02/10/2025

Electronic publication
