# Supplementary material for: Age-related TFEB downregulation in proximal tubules causes systemic metabolic disorders and occasional apolipoprotein A4–related amyloidosis
Source: JCI Insight. 2024 Dec 19;10(3):e184451. doi: 10.1172/jci.insight.184451 (PMC11948592; doi:10.1172/jci.insight.184451)
Supplement: Supplemental data [file jciinsight-10-184451-s089.pdf]

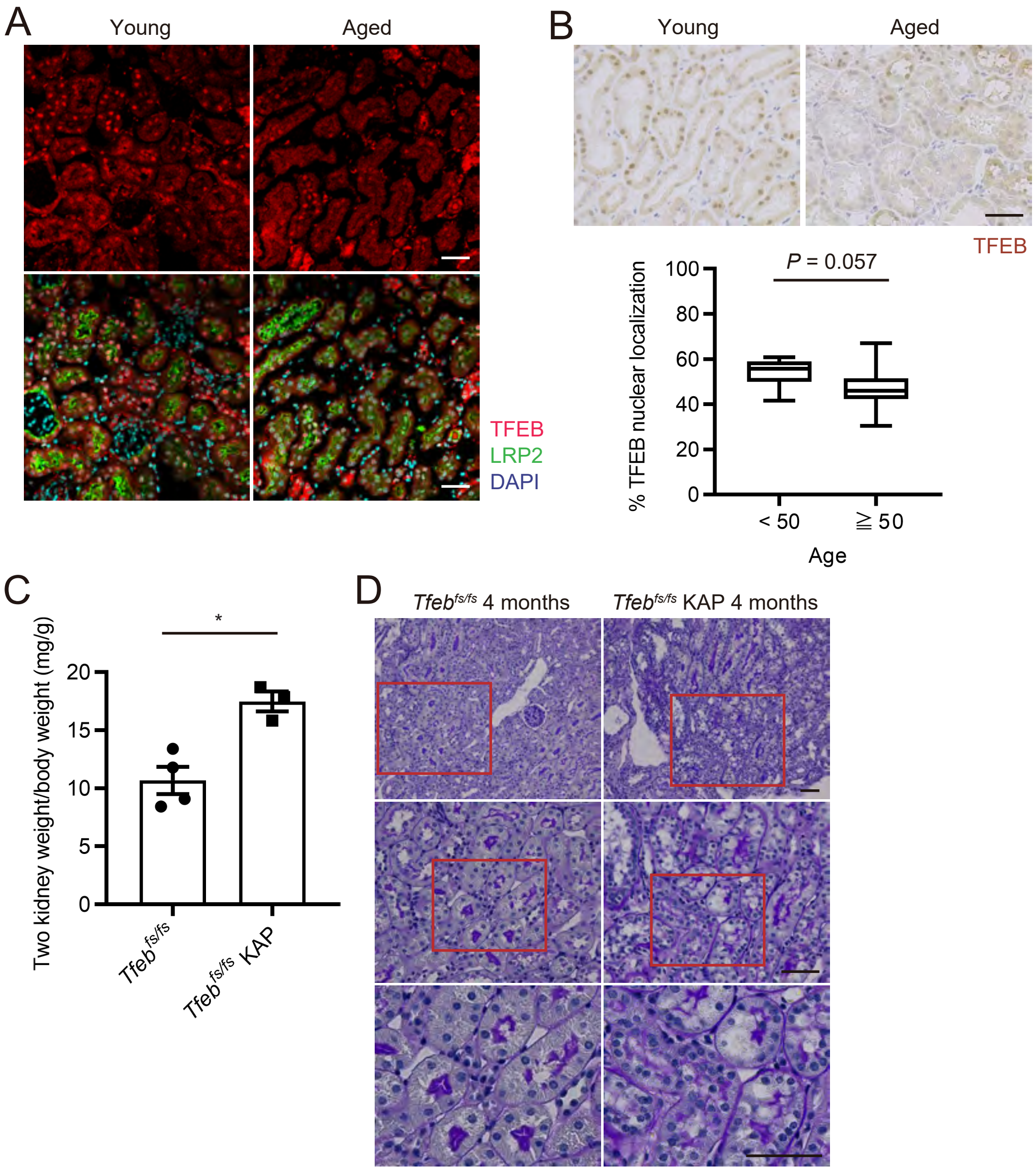

Supplemental Figure 1. PTEC-specific *Tfeb*-overexpressing mice shows aberrant cell proliferation. (**A**) Representative low-magnification images showing TFEB in the kidney cortical regions of young (6-week-old) or aged (2-year-old) mice (n = 11). Sections were immunostained for LRP2, a marker of proximal tubules (green) and counterstained with DAPI (blue). (**B**) Representative images of immunohistochemical staining for TFEB on kidney specimens obtained from young (under 50 years old) or aged (over 50 years old) patients. Specimens were counterstained with hematoxylin. Kidney biopsy samples from CKD patients were analyzed, using the dataset previously reported in our papers (ref. 20 (n = 21)). (**C**) The ratio of the combined kidney weight to the total body weight of 4-month-old $Tfeb^{fs/fs}$ or $Tfeb^{fs/fs}$ KAP mice (n = 3–4). (**D**) Representative images of PAS staining in the kidneys of 4-month-old $Tfeb^{fs/fs}$ or $Tfeb^{fs/fs}$ KAP mice (n = 3–4). Magnified images in $Tfeb^{fs/fs}$ KAP mice show aberrant cell proliferation and vacuolar degeneration in proximal tubules. Bars: 40 µm (**A**) and 50 µm (**B** and **D**). Data are provided as bar graphs showing means ± standard error (SE) or box plots showing median values and interquartile range. Boxes represent the 25th and 75th percentile, lines inside the boxes represent medians, whiskers are plotted by Tukey method. Statistically significant differences: $^{*}P < 0.05$ versus $Tfeb^{fs/fs}$ control littermates (**B**, Wilcoxon rank sum test; **C**, two-tailed Student’ s *t* test). $Tfeb^{fs/fs}$, mice carrying $Tfeb\text{-}3xFlag^{fs/fs}$ under the control of a strong CAG promoter; $Tfeb^{fs/fs}$ KAP, mice carrying $Tfeb\text{-}3xFlag^{fs/fs}$ under the control of a strong CAG promoter crossed with the KAP–Cre.

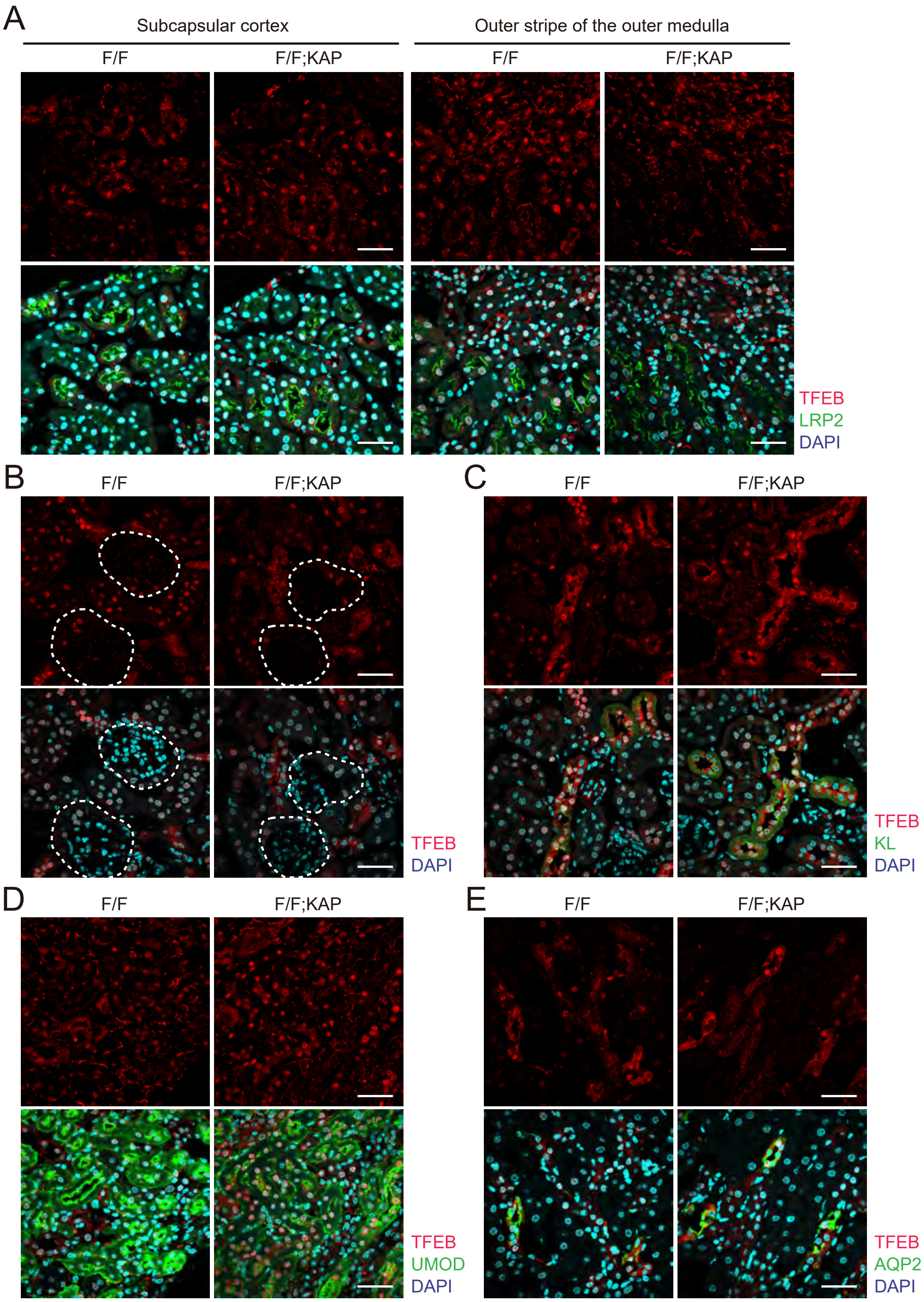
A
Subcapsular cortex
Outer stripe of the outer medulla
F/F
F/F;KAP
F/F
F/F;KAP
TFEB
LRP2
DAPI
B
F/F
F/F;KAP
TFEB
DAPI
C
F/F
F/F;KAP
TFEB
KL
DAPI
D
F/F
F/F;KAP
TFEB
UMOD
DAPI
E
F/F
F/F;KAP
TFEB
AQP2
DAPI

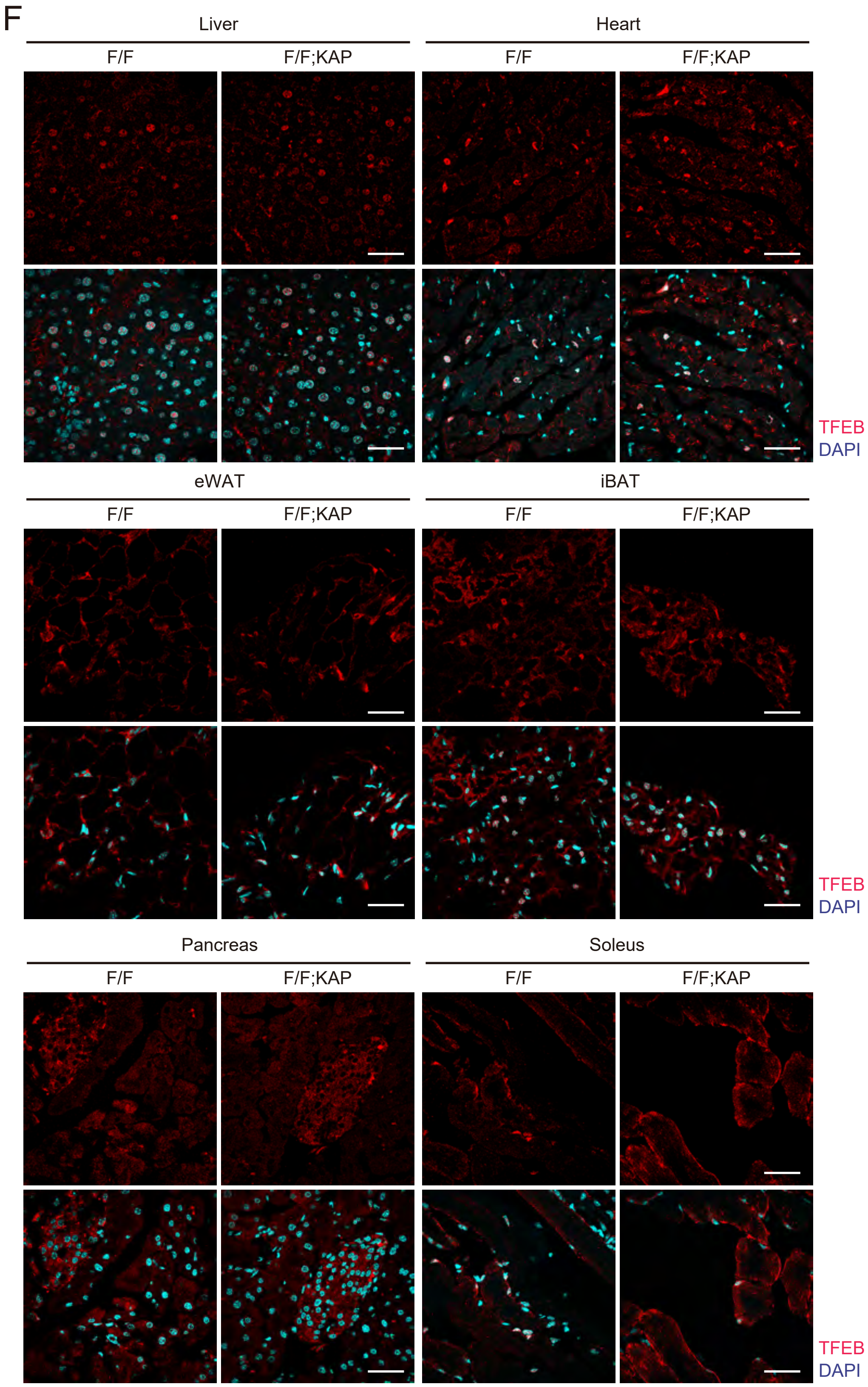

Supplemental Figure 2. TFEB is specifically deleted in the S3 segment of the proximal tubules. (**A**–**E**) Representative immunofluorescence images showing TFEB in different segments of the kidneys of young mice. Sections were coimmunostained with LRP2 (a marker of the proximal tubules) (**A**), KL (mainly expressed in the distal tubules) (**C**), UMOD (mainly expressed in the thick ascending limbs of the loop of Henle) (**D**), or AQP2 (a marker of the collecting ducts) (**E**). S1/S2 segments are mainly disributed in the subcapsular cortex, while S3 segments are mainly disributed in the outer stripe of the outer medulla (**A**). White dotted lines enclose renal corpuscles, which consist of glomuruli and Bowman's capsules (**B**). (**F**) Representative immunofluorescence images showing TFEB in different organs. Sections were counterstained with DAPI. Bars: 40 μm. F/F, *Tfeb*$^{fl/fl}$ mice; F/F;KAP, *Tfeb*$^{fl/fl}$ KAP mice.

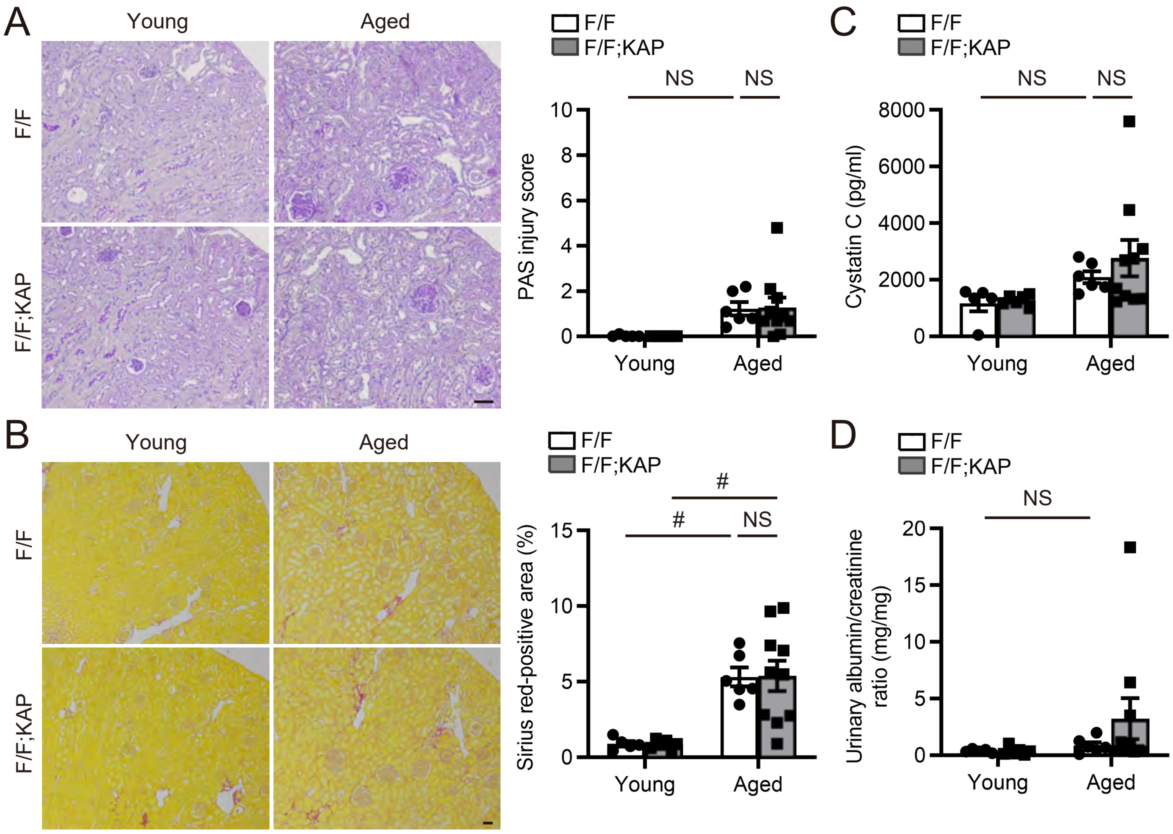

Supplemental Figure 3. Long-term TFEB deficiency in PTECs does not largely induce renal dysfunction. (**A** and **B**) Representative images of PAS staining (**A**) and picrosirius red staining (**B**) in the kidneys of young (6-week-old) and aged (2-year-old) $Tfeb^{fl/fl}$ or $Tfeb^{fl/fl}$ KAP mice (n = 5–10). The tubular injury score and the sirius red-positive area was shown. (**C** and **D**) Plasma cystatin C concentrations and urinary albumin/creatinine ratio in the young and aged $Tfeb^{fl/fl}$ or $Tfeb^{fl/fl}$ KAP mice (n = 5–10). Bars: 50 µm. Data are provided as bar graphs showing means ± standard error (SE). Statistically significant differences: $^{\#}P < 0.05$ versus young mice (one-way ANOVA followed by the Tukey-Kramer test). F/F, $Tfeb^{fl/fl}$ mice; F/F;KAP, $Tfeb^{fl/fl}$ KAP mice.

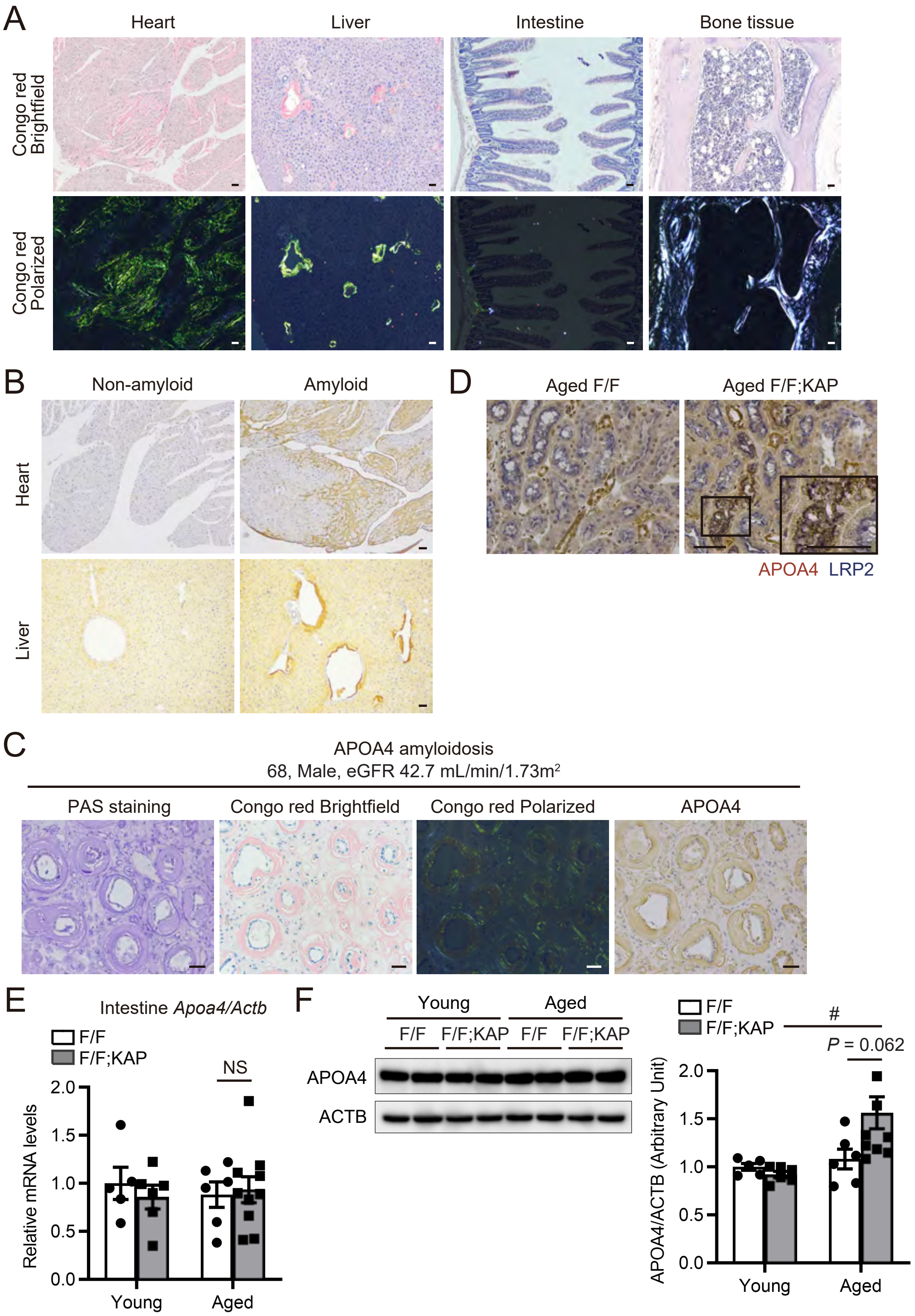

Supplemental Figure 4. TFEB deficiency in PTECs causes systemic APOA4 amyloidosis with age. (**A** and **B**) Representative images of Congo red staining (**A**) and APOA4 immunostaining (**B**) in various organs of aged (2-year-old) *Tfeb$^{fl/fl}$* KAP mice with (**A** and **B**) or without (**B**) amyloid deposits. Sections were counterstained with hematoxylin. (**C**) Representative images of PAS staining, Congo red staining, and immunohistochemical staining for APOA4 in the medulla regions of human autopsy kidney samples. Specimens were counterstained with hematoxylin. (**D**) Representative images of APOA4 immunostaining in the kidneys of aged *Tfeb$^{fl/fl}$* or *Tfeb$^{fl/fl}$* KAP mice. Sections were immunostained for LRP2, a marker of proximal tubules (blue) and counterstained with hematoxylin. (**E**) *Apoa4* mRNA levels relative to *Actb* in the intestine of young and aged *Tfeb$^{fl/fl}$* or *Tfeb$^{fl/fl}$* KAP mice (n = 5–10). (**F**) Representative western blot images of APOA4 in liver lysates of young and aged *Tfeb$^{fl/fl}$* or *Tfeb$^{fl/fl}$* KAP mice (n = 5–10). Blots were set up in parallel and run contemporaneously. Values are normalized by the mean value of young *Tfeb$^{fl/fl}$* mice. Statistically significant differences: $^{\#}P < 0.05$ versus young mice (**E** and **F**, 1-way ANOVA followed by the Tukey-Kramer test). Bars: 50 μm (**A**–**D**). Data are provided as bar graphs showing means ± standard error (SE). Bright field images of Congo red staining were captured along with corresponding apple green birefringence under polarized light. F/F, *Tfeb$^{fl/fl}$* mice; F/F;KAP, *Tfeb$^{fl/fl}$* KAP mice; eGFR, estimated glomerular filtration rate.

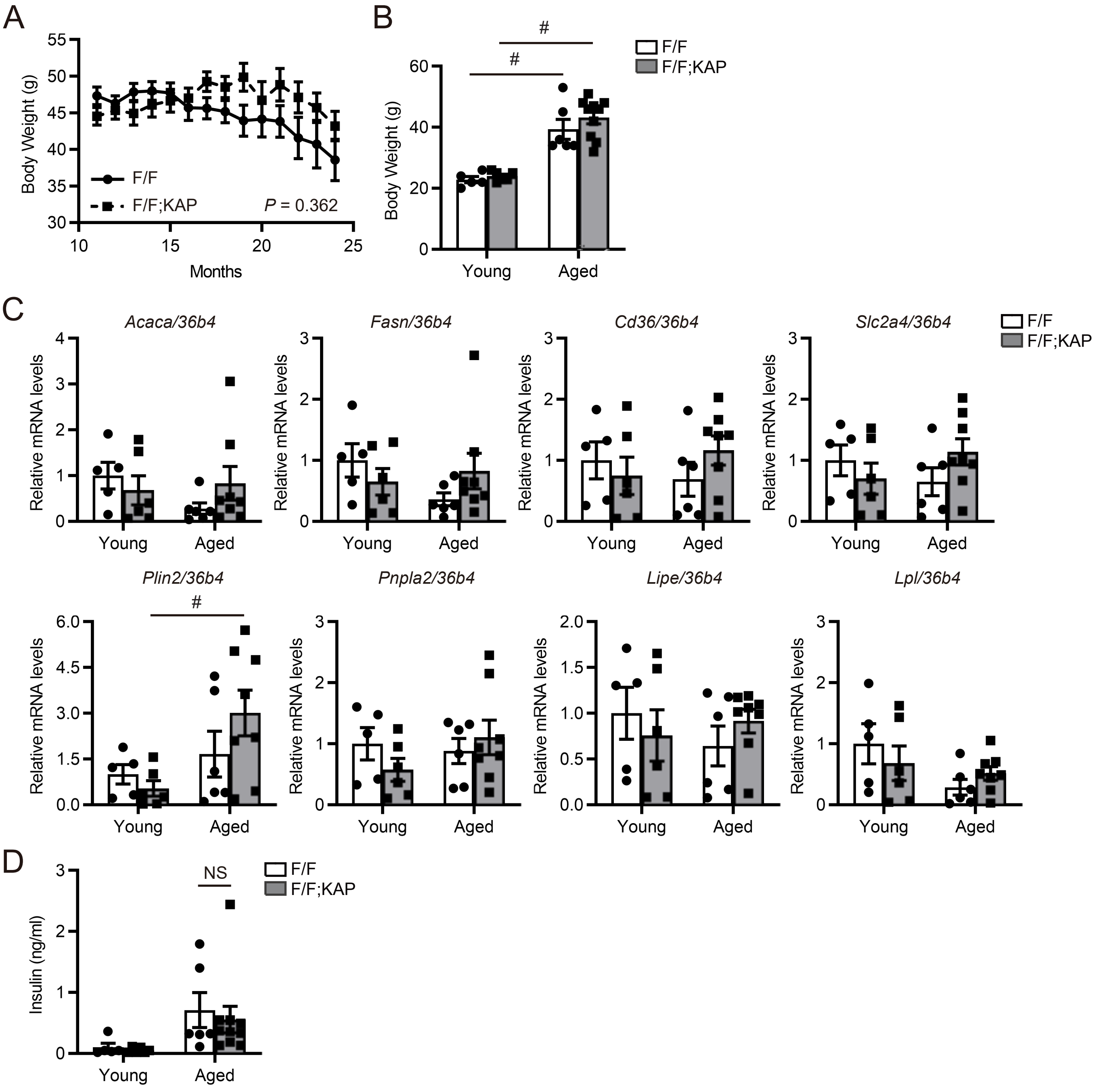

Supplemental Figure 5. TFEB deficiency in PTECs increases adipogenesis in aged mice. (**A**) Body weight chart for *Tfeb*$^{fl/fl}$ (n = 13) or *Tfeb*$^{fl/fl}$ KAP mice (n = 15). (**B**) Body weight at terminal sacrifice of young (6-week-old) and aged (2-year-old) *Tfeb*$^{fl/fl}$ or *Tfeb*$^{fl/fl}$ KAP mice (n = 5–10). (**C**) mRNA levels relative to *36b4* in epididymal white adipose tissue of young and aged *Tfeb*$^{fl/fl}$ or *Tfeb*$^{fl/fl}$ KAP mice (n = 5–8). Values are normalized by the mean value of young *Tfeb*$^{fl/fl}$ mice. (**D**) Plasma insulin levels of young and aged *Tfeb*$^{fl/fl}$ or *Tfeb*$^{fl/fl}$ KAP mice (n = 5–10). Data are provided as bar graphs showing means ± standard error (SE). Statistically significant differences: $^{\#}P < 0.05$ versus young littermates (one-way ANOVA followed by the Tukey-Kramer test). Mixed-effects analysis was used to assess body weight progress. F/F, *Tfeb*$^{fl/fl}$ mice; F/F;KAP, *Tfeb*$^{fl/fl}$ KAP mice.

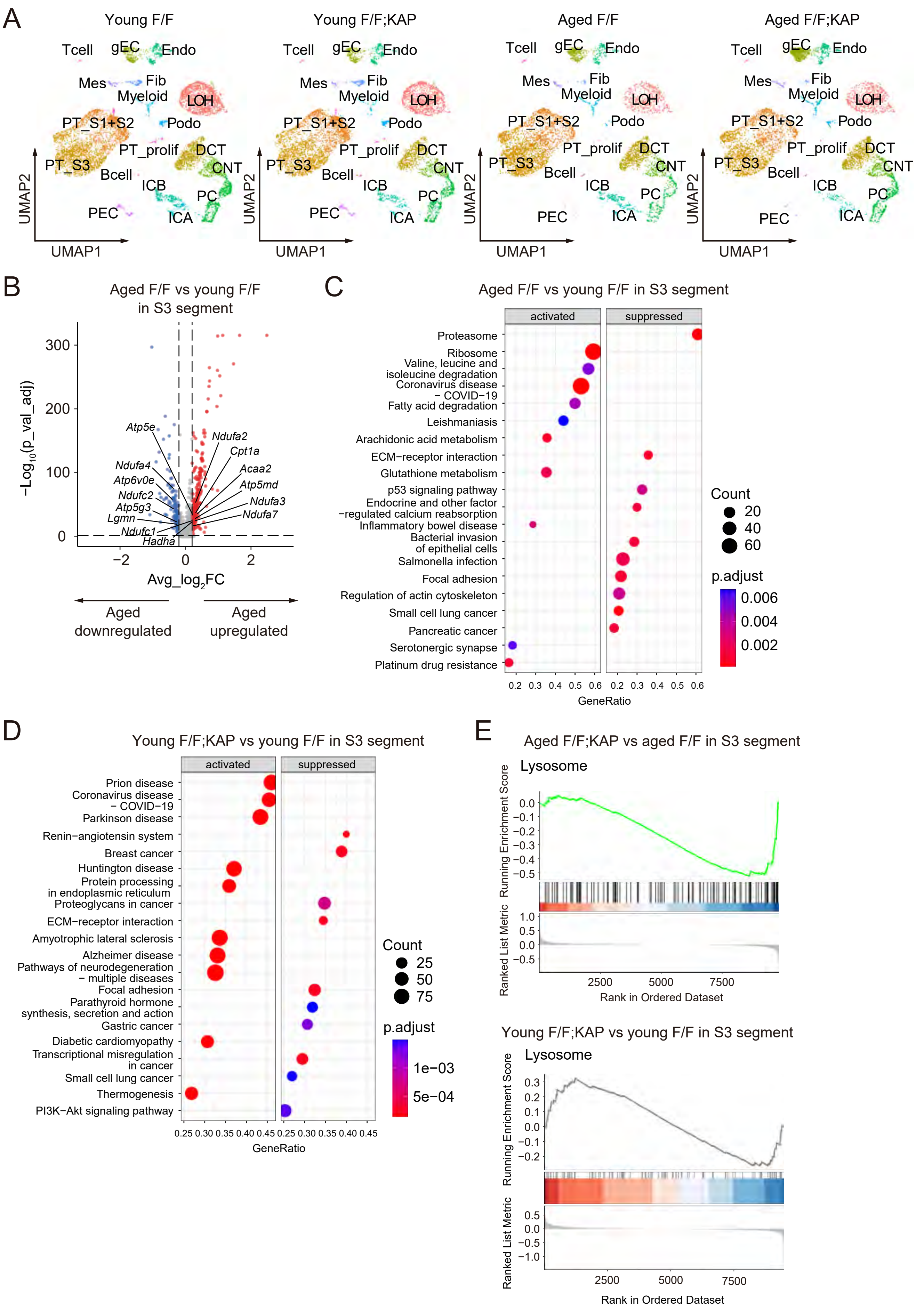
A
Young F/F
Young F/F;KAP
Aged F/F
Aged F/F;KAP
Tcell
gEC
Endo
Mes
Fib
Myeloid
LOH
PT_S1+S2
Podo
PT_prolif
DCT
PT_S3
CNT
Bcell
ICB
PC
PEC
ICA
UMAP2
UMAP1
B
Aged F/F vs young F/F
in S3 segment
−Log10(p_val_adj)
300
200
100
0
−2
0
2
Avg_log2FC
Atp5e
Ndufa4
Atp6v0e
Ndufc2
Atp5g3
Lgmn
Ndufc1
Hadha
Ndufa2
Cpt1a
Acaa2
Atp5md
Ndufa3
Ndufa7
Aged
downregulated
Aged
upregulated
C
Aged F/F vs young F/F in S3 segment
activated
suppressed
Proteasome
Ribosome
Valine, leucine and
isoleucine degradation
Coronavirus disease
− COVID−19
Fatty acid degradation
Leishmaniasis
Arachidonic acid metabolism
ECM−receptor interaction
Glutathione metabolism
p53 signaling pathway
Endocrine and other factor
−regulated calcium reabsorption
Inflammatory bowel disease
Bacterial invasion
of epithelial cells
Salmonella infection
Focal adhesion
Regulation of actin cytoskeleton
Small cell lung cancer
Pancreatic cancer
Serotonergic synapse
Platinum drug resistance
0.2 0.3 0.4 0.5 0.6
0.2 0.3 0.4 0.5 0.6
GeneRatio
Count
20
40
60
p.adjust
0.006
0.004
0.002
D
Young F/F;KAP vs young F/F in S3 segment
activated
suppressed
Prion disease
Coronavirus disease
− COVID−19
Parkinson disease
Renin−angiotensin system
Breast cancer
Huntington disease
Protein processing
in endoplasmic reticulum
Proteoglycans in cancer
ECM−receptor interaction
Amyotrophic lateral sclerosis
Alzheimer disease
Pathways of neurodegeneration
− multiple diseases
Focal adhesion
Parathyroid hormone
synthesis, secretion and action
Gastric cancer
Diabetic cardiomyopathy
Transcriptional misregulation
in cancer
Small cell lung cancer
Thermogenesis
PI3K−Akt signaling pathway
0.25 0.30 0.35 0.40 0.45
0.25 0.30 0.35 0.40 0.45
GeneRatio
Count
25
50
75
p.adjust
1e−03
5e−04
E
Aged F/F;KAP vs aged F/F in S3 segment
Lysosome
Running Enrichment Score
0.0
−0.1
−0.2
−0.3
−0.4
−0.5
Ranked List Metric
1.0
0.5
0.0
−0.5
2500
5000
7500
Rank in Ordered Dataset
Young F/F;KAP vs young F/F in S3 segment
Lysosome
Running Enrichment Score
0.3
0.2
0.1
0.0
−0.1
−0.2
Ranked List Metric
0.5
0.0
−0.5
−1.0
2500
5000
7500
Rank in Ordered Dataset

F

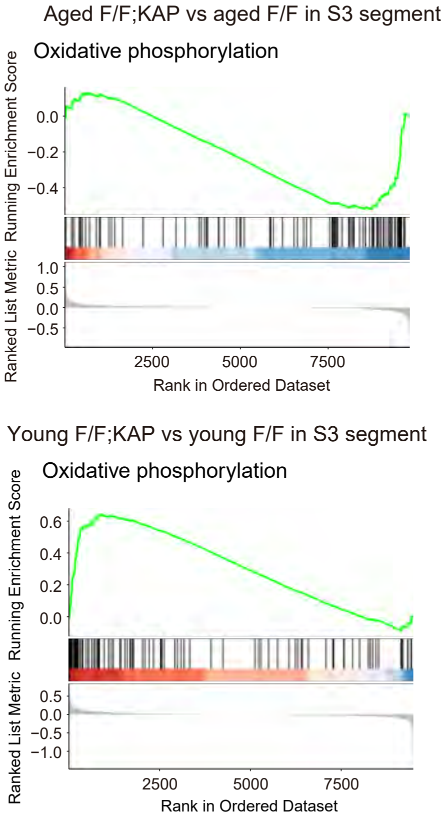

G

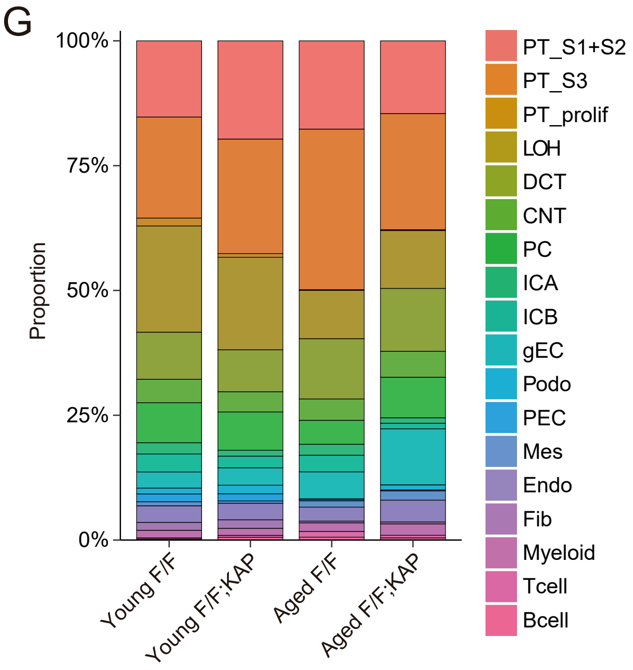

Supplemental Figure 6. TFEB deficiency does not cause downregulation of lysosome or oxidative phosphorylation pathway in the S3 segment of young mice. (**A**) Cells in each group were mapped by UMAP separately. (**B**) Volcano plots depicting differentially expressed genes. Plots were discriminated based on adjusted *P* value < 0.05 and $\log_2$ fold change threshold = 0.20. Colored dots correspond to individual genes whose expression levels were downregulated (blue) and upregulated (red). (**C** and **D**) Results of KEGG pathway gene set enrichment analyses using the gseKEGG in the R package clusterProfiler. The dot size represents the numbers of genes. The dot color scale corresponds to the adjusted *P* value. (**E** and **F**) GSEA-based KEGG-enrichment plots show that the "Lysosome" and "Oxidative phosphorylation" pathway in S3 segment were downregulated not in young *Tfeb*$^{fl/fl}$ KAP mice, but in aged *Tfeb*$^{fl/fl}$ KAP mice. The running enrichment score is plotted as a function of the position in the ranked list of genes. (**G**) Bar plots showing the relative proportion of each cluster. p_val_ad, adjusted *P* value; Avg_log$_2$FC, average $\log_2$ fold change; F/F, *Tfeb*$^{fl/fl}$ mice; F/F;KAP, *Tfeb*$^{fl/fl}$ KAP mice.

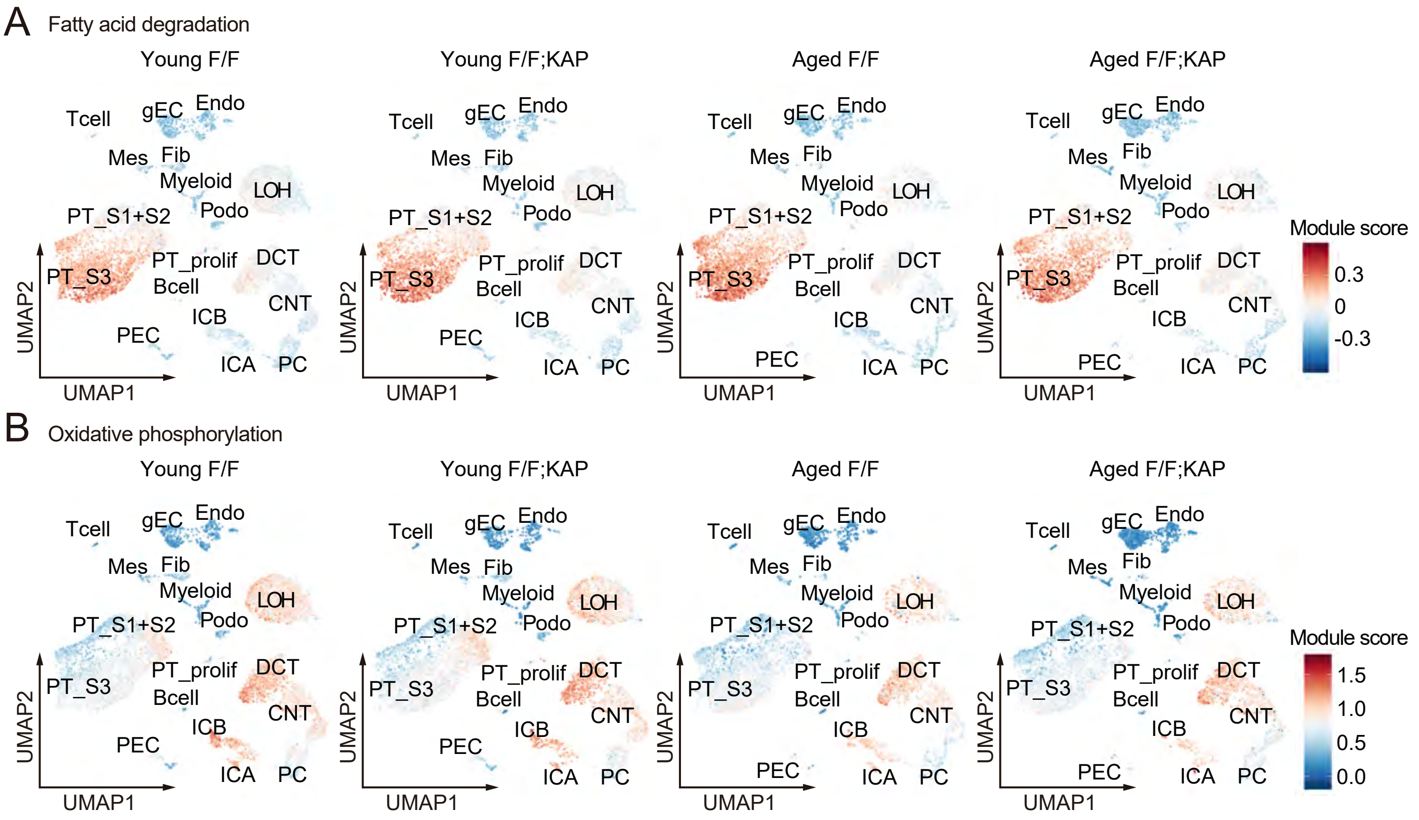

Supplemental Figure 7. Module score distribution. (**A** and **B**) The module score distribution in UMAP using "Fatty acid degradation" and "Oxidative phosphorylation" gene sets. F/F, *Tfeb*$^{fl/fl}$ mice; F/F;KAP, *Tfeb*$^{fl/fl}$ KAP mice.

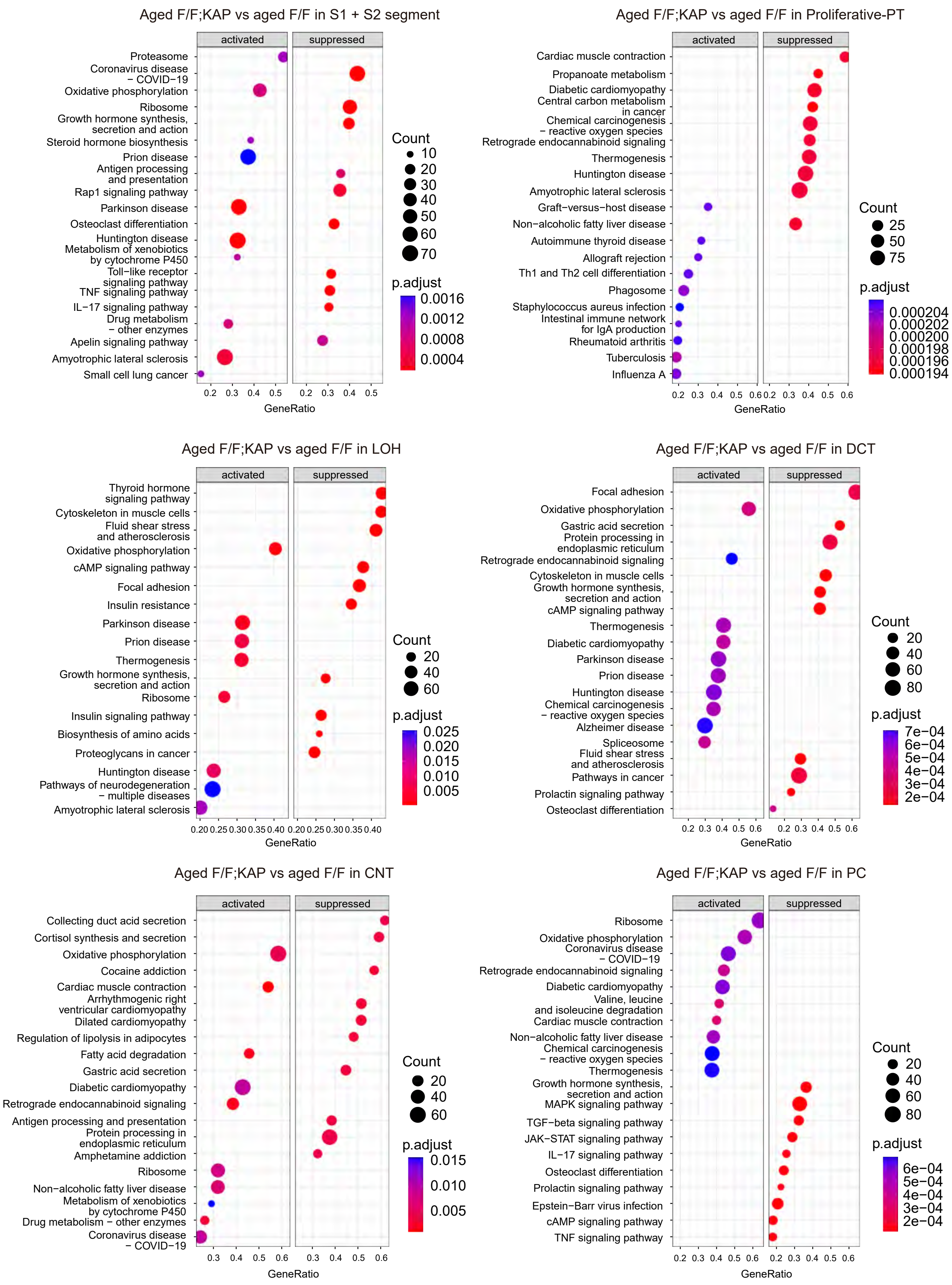

Aged F/F;KAP vs aged F/F in S1 + S2 segment
activated
suppressed
Proteasome
Coronavirus disease − COVID−19
Oxidative phosphorylation
Ribosome
Growth hormone synthesis, secretion and action
Steroid hormone biosynthesis
Prion disease
Antigen processing and presentation
Rap1 signaling pathway
Parkinson disease
Osteoclast differentiation
Huntington disease
Metabolism of xenobiotics by cytochrome P450
Toll−like receptor signaling pathway
TNF signaling pathway
IL−17 signaling pathway
Drug metabolism − other enzymes
Apelin signaling pathway
Amyotrophic lateral sclerosis
Small cell lung cancer
GeneRatio
Count
10
20
30
40
50
60
70
p.adjust
0.0016
0.0012
0.0008
0.0004
Aged F/F;KAP vs aged F/F in Proliferative-PT
Cardiac muscle contraction
Propanoate metabolism
Diabetic cardiomyopathy
Central carbon metabolism in cancer
Chemical carcinogenesis − reactive oxygen species
Retrograde endocannabinoid signaling
Thermogenesis
Huntington disease
Amyotrophic lateral sclerosis
Graft−versus−host disease
Non−alcoholic fatty liver disease
Autoimmune thyroid disease
Allograft rejection
Th1 and Th2 cell differentiation
Phagosome
Staphylococcus aureus infection
Intestinal immune network for IgA production
Rheumatoid arthritis
Tuberculosis
Influenza A
Count
25
50
75
p.adjust
0.000204
0.000202
0.000200
0.000198
0.000196
0.000194
Aged F/F;KAP vs aged F/F in LOH
Thyroid hormone signaling pathway
Cytoskeleton in muscle cells
Fluid shear stress and atherosclerosis
Oxidative phosphorylation
cAMP signaling pathway
Focal adhesion
Insulin resistance
Parkinson disease
Prion disease
Thermogenesis
Growth hormone synthesis, secretion and action
Ribosome
Insulin signaling pathway
Biosynthesis of amino acids
Proteoglycans in cancer
Huntington disease
Pathways of neurodegeneration − multiple diseases
Amyotrophic lateral sclerosis
Count
20
40
60
p.adjust
0.025
0.020
0.015
0.010
0.005
Aged F/F;KAP vs aged F/F in DCT
Focal adhesion
Oxidative phosphorylation
Gastric acid secretion
Protein processing in endoplasmic reticulum
Retrograde endocannabinoid signaling
Cytoskeleton in muscle cells
Growth hormone synthesis, secretion and action
cAMP signaling pathway
Thermogenesis
Diabetic cardiomyopathy
Parkinson disease
Prion disease
Huntington disease
Chemical carcinogenesis − reactive oxygen species
Alzheimer disease
Spliceosome
Fluid shear stress and atherosclerosis
Pathways in cancer
Prolactin signaling pathway
Osteoclast differentiation
Count
20
40
60
80
p.adjust
7e−04
6e−04
5e−04
4e−04
3e−04
2e−04
Aged F/F;KAP vs aged F/F in CNT
Collecting duct acid secretion
Cortisol synthesis and secretion
Oxidative phosphorylation
Cocaine addiction
Cardiac muscle contraction
Arrhythmogenic right ventricular cardiomyopathy
Dilated cardiomyopathy
Regulation of lipolysis in adipocytes
Fatty acid degradation
Gastric acid secretion
Diabetic cardiomyopathy
Retrograde endocannabinoid signaling
Antigen processing and presentation
Protein processing in endoplasmic reticulum
Amphetamine addiction
Ribosome
Non−alcoholic fatty liver disease
Metabolism of xenobiotics by cytochrome P450
Drug metabolism − other enzymes
Coronavirus disease − COVID−19
Count
20
40
60
p.adjust
0.015
0.010
0.005
Aged F/F;KAP vs aged F/F in PC
Ribosome
Oxidative phosphorylation
Coronavirus disease − COVID−19
Retrograde endocannabinoid signaling
Diabetic cardiomyopathy
Valine, leucine and isoleucine degradation
Cardiac muscle contraction
Non−alcoholic fatty liver disease
Chemical carcinogenesis − reactive oxygen species
Thermogenesis
Growth hormone synthesis, secretion and action
MAPK signaling pathway
TGF−beta signaling pathway
JAK−STAT signaling pathway
IL−17 signaling pathway
Osteoclast differentiation
Prolactin signaling pathway
Epstein−Barr virus infection
cAMP signaling pathway
TNF signaling pathway
Count
20
40
60
80
p.adjust
6e−04
5e−04
4e−04
3e−04
2e−04

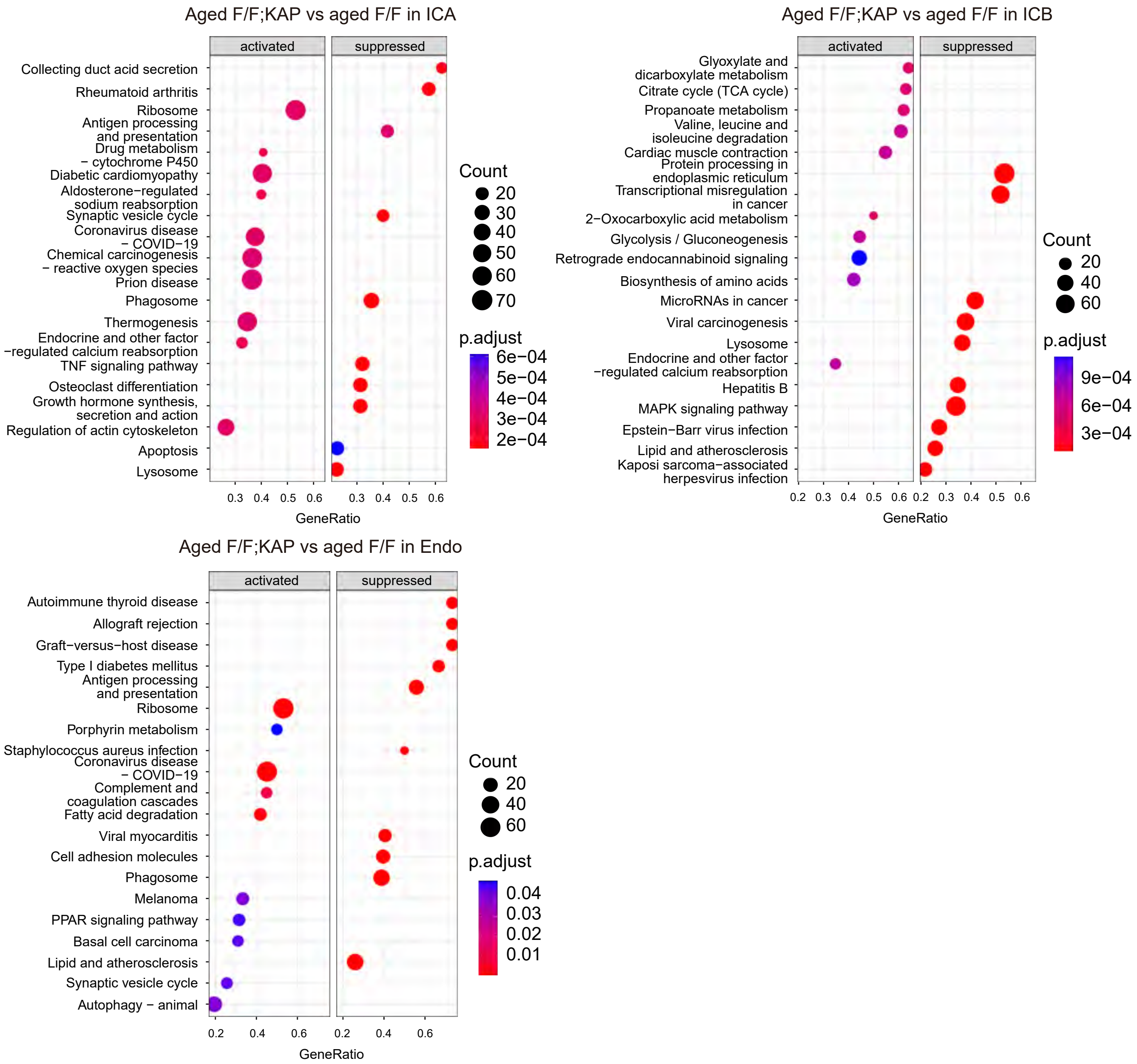

Supplemental Figure 8. KEGG pathway gene set enrichment analyses. Results of KEGG pathway gene set enrichment analyses in each cluster other than S3 segment, using the gseKEGG in the R package clusterProfiler. The dot size represents the numbers of genes. The dot color scale corresponds to the adjusted *P* value. F/F, *Tfeb*$^{fl/fl}$ mice; F/F;KAP, *Tfeb*$^{fl/fl}$ KAP mice.

```
sp|P06728| MFLKAAVLTLALVAITGTRAEVTSDQVANVVWDYFTQLSNNAKEAVEQFQKTDVTQQLST
sp|P06727| MFLKAVVLTLALVAVAGARAEVSADQVATVMWDYFSQLSNNAKEAVEHLQKSELTQQLNA
AEX20370.1 ------------------------------------------------------------

sp|P06728| LFQDKLGDASTYADGVHNKLVPFVVQLSGHLAQETERVKEEIKKELEDLRDRMMPHANKV
sp|P06727| LFQDKLGEVNTYAGDLQKKLVPFATELHERLAKDSEKLKEEIGKELEELRARLLPHANEV
AEX20370.1 ----------------------FATELHERLAKDSEKLKEEIRKELEEVRARLLPHASEV

sp|P06728| TQTFGENMQKLQEHLKPYAVDLQDQINTQTQEMKLQLTPYIQRMQTTIKENVDNLHTSMM
sp|P06727| SQKIGDNLRELQQRLEPYADQLRTQVSTQAEQLRRQLTPYAQRMERVLRENADSLQASLR
AEX20370.1 SQKIGDNVRELQQRLGPYADELRTQVNTQAEQLRRQLTPYAQRMQRVLRENADSLQSSLK

sp|P06728| PLATNLKDKFNRNMEELKGHLTPRANELKATIDQNLEDLRRSLAPLTVGVQEKLNHQMEG
sp|P06727| PHADELKAKIDQNVEELKGRLTPYADEFKVKIDQTVEELRRSLAPYAQDTQEKLNHQLEG
AEX20370.1 PHADELKAKIDQNVEELKGRLTPYADELKVKIDQTVEELRHSLAPYAQDAQEKLNHQLEG

sp|P06728| LAFQMKKNAEELQTKVSAKIDQLQKNLAPLVEDVQSKVKGNTEGLQKSLEDLNRQLEQQV
sp|P06727| LTFQMKKNAEELKARISASAEELRQRLAPLAEDVRGNLRGNTEGLQKSLAELGGHLDQQV
AEX20370.1 LAFQMKKNAEELKARISANAEELRQRLAPVAEDVHGNLRANTEELQKSLAELGGHLDRQV

sp|P06728| EEFRRTVEPMGEMFNKALVQQLEQFRQQLGPNSGEVESHLSFLEKSLREKVNSFMSTLEK
sp|P06727| EEFRRRVEPYGENFNKALVQQMEQLRQKLGPHAGDVEGHLSFLEKDLRDKVNSFFSTFKE
AEX20370.1 EEFRRQVEPYGESFNKALVQQMEQLRQKLGPHAGDMEGHLSFLEKD--------------

sp|P06728| KGSPDQPQALPLPEQAQEQAQEQAQEQVQP-KPLES
sp|P06727| KESQDKTLSLPELEQQQEQQQEQQQEQVQMLAPLES
AEX20370.1 ------------------------------------
```

Supplemental Figure 9. APOA4-derived peptides identified by proteomics. APOA4-derived peptides detected from this mice study were highlighted in yellow (UniProt accession: P06728). Peptides identified in other human and monkey studies (ref.48 and 79) were also highlighted in green and blue, respectively (UniProt accession: P06727 and NCBI accession: AEX20370.1, respectively). Red underline shows APOA4 signal sequence reported in ref.74.
