# Supplementary material for: Age-related TFEB downregulation in proximal tubules causes systemic metabolic disorders and occasional apolipoprotein A4–related amyloidosis
Source: JCI Insight. 2024 Dec 19;10(3):e184451. doi: 10.1172/jci.insight.184451 (PMC11948592; doi:10.1172/jci.insight.184451)

Full unedited gel for Figure 1C

p-S6RP

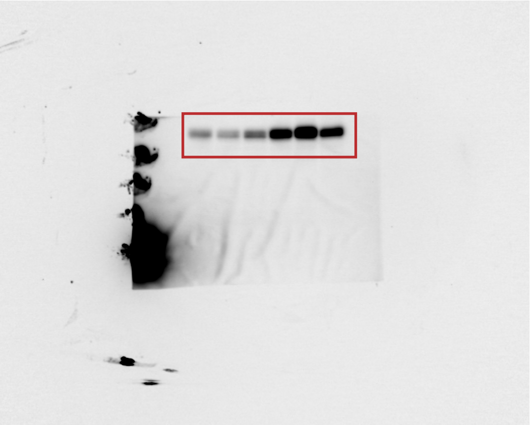

S6RP

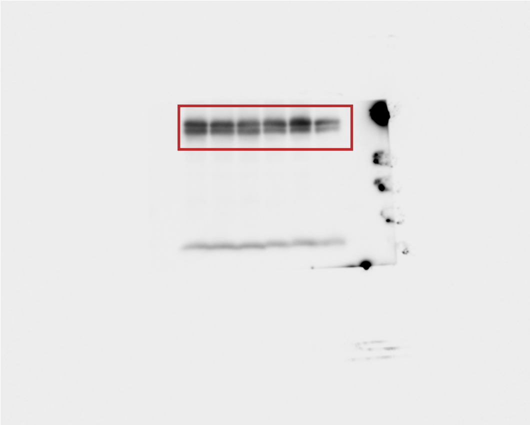

ACTB

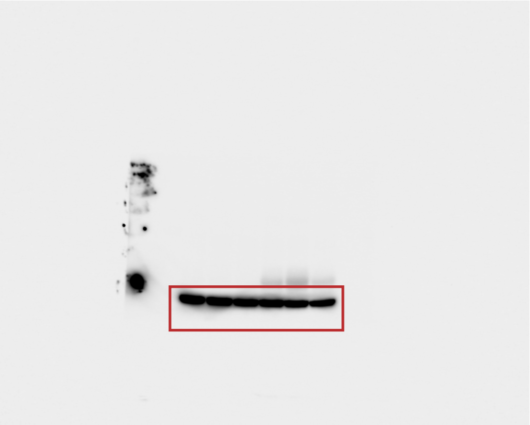

Full unedited gel for Figure 2G

His-Tag

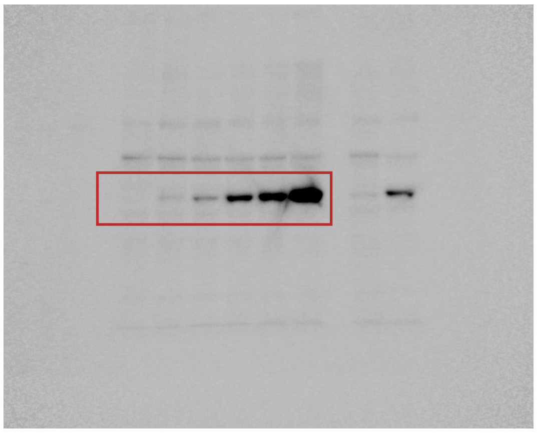

GAPDH

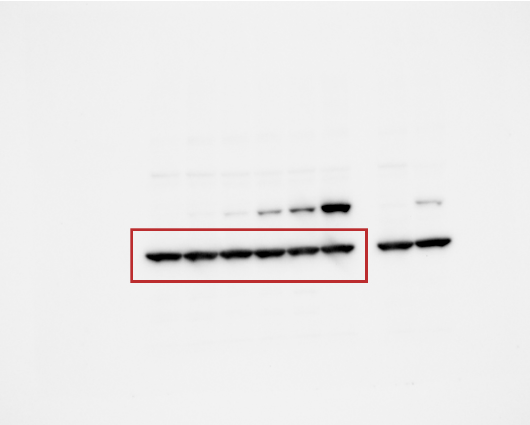

Full unedited gel for Figure 2H

His-Tag

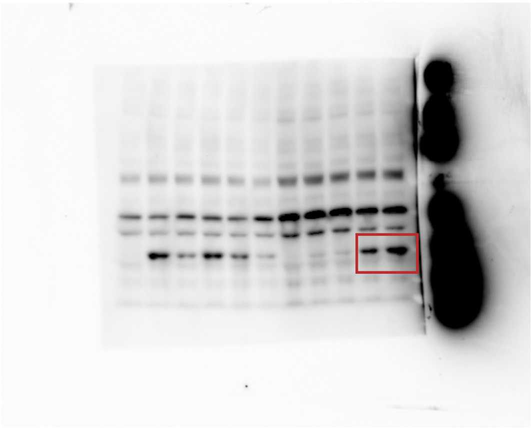

TFEB

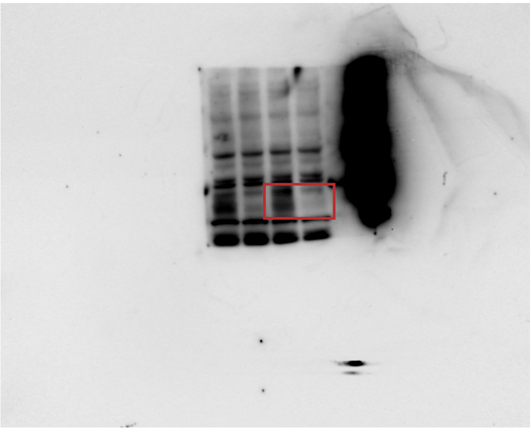

GAPDH

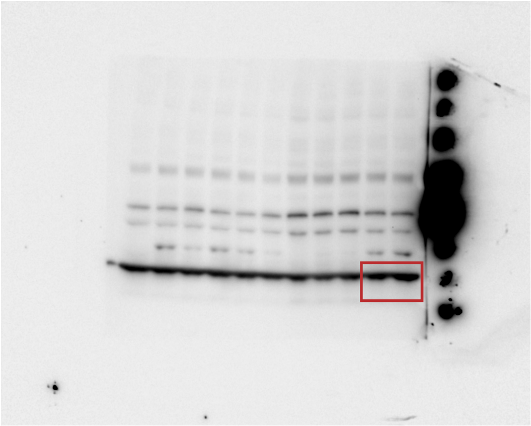

Full unedited gel for Supplemental Figure 4F

APOA4

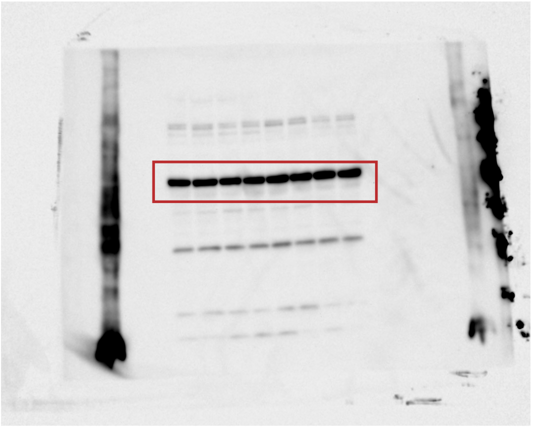

ACTB

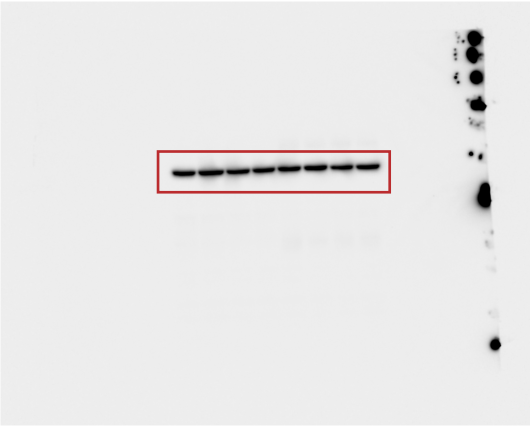

Supplement: Unedited blot and gel images [file jciinsight-10-184451-s090.pdf]
